# The master virulence regulator PhoP dictates carbon metabolism by controlling cyclic AMP synthesis in *Salmonella*

**DOI:** 10.1371/journal.pbio.3003566

**Published:** 2025-12-18

**Authors:** Nick D. Pokorzynski, Elisabeth C. Sams-Dodd, Christopher Esneault, Katarina A. Jones, Shawn R. Campagna, Eduardo A. Groisman

**Affiliations:** 1 Department of Microbial Pathogenesis, Yale School of Medicine, New Haven, Connecticut, United States of America; 2 Biological and Small Molecule Mass Spectrometry Core, University of Tennessee, Knoxville, Tennessee, United States of America; 3 Department of Chemistry, University of Tennessee, Knoxville, Tennessee, United States of America; University of California Davis School of Medicine, UNITED STATES OF AMERICA

## Abstract

The intracellular pathogen *Salmonella enterica* serovar Typhimurium confronts cytoplasmic Mg^2+^ starvation inside macrophages. This stress alters carbon metabolism and subverts canonical carbon source preferences by reducing synthesis of 3′, 5′-cyclic adenosine monophosphate (cAMP), the essential allosteric activator of the cAMP receptor protein (CRP), master regulator of carbon utilization. How, then, does *S.* Typhimurium preferentially utilize CRP-cAMP-dependent carbon sources inside macrophages? We now report that the virulence and Mg^2+^ homeostasis regulator PhoP controls CRP-cAMP-dependent transcription, metabolism, and growth on a mixture of carbon sources during low cytoplasmic Mg^2+^. We determine that the PhoP-activated MgtA and MgtB proteins promote CRP-cAMP activity by importing Mg^2+^, indispensable cofactor of the cAMP-synthesizing adenylate cyclase CyaA. Significantly, the PhoP-activated MgtC preserves cAMP amounts despite reducing abundance of CyaA substrate adenosine triphosphate (ATP) because ATP at high concentrations inhibits CyaA. Restoring CRP activity by supplementation of cAMP or introduction of the constitutively active crp* allele corrected CRP-dependent transcriptional and growth behaviors of the *mgtA mgtB* mutant. By controlling cAMP synthesis, PhoP dictates the amounts of active CRP, thereby reprogramming *S.* Typhimurium’s metabolism.

## Abstract 2

Inside macrophages, Salmonella faces magnesium scarcity that disrupts cAMP synthesis and carbon metabolism. This study shows that the PhoP regulator restores metabolic control by promoting Mg2+ uptake and directing cAMP production to sustain growth via the cAMP receptor protein (CRP).

## Introduction

In all organisms, carbon source governs metabolism. While organisms differ in carbon source preference, many bacterial species, including the gastroenteritis- and murine typhoid fever-causing *Salmonella enterica* serovar Typhimurium, choose glucose over other carbon sources, such as glycerol and gluconate, when grown in laboratory media [1]. Surprisingly, *S.* Typhimurium displays the opposite preference inside mammalian macrophages [2–4], where *S.* Typhimurium experiences cytoplasmic Mg^2+^ starvation [5,6], a stress that reduces protein synthesis and slows bacterial growth [7,8]. Cytoplasmic Mg^2+^ starvation supersedes *S.* Typhimurium’s canonical carbon source preference by reducing the abundance of 3′, 5′-cyclic adenosine monophosphate (cAMP) [2], the allosteric activator of the major regulator of carbohydrate utilization, the cAMP receptor protein (CRP) [9], sharply suppressing its activity [2]. Here, we establish that the master regulator of *S.* Typhimurium virulence and Mg^2+^ homeostasis governs metabolism by controlling cAMP synthesis.

*S.* Typhimurium and other enteric bacteria harbor a regulatory system—termed PhoP/PhoQ—that governs virulence and Mg^2+^ homeostasis [10]. The sensor PhoQ activates the DNA-binding protein PhoP in response to low extracytoplasmic Mg^2+^ as well as other signals [11–13]. PhoP promotes transcription of hundreds of genes [10], including *mgtA* and *mgtB*, which specify P-type ATPases that import Mg^2+^ from the periplasm into the cytoplasm [14], and *mgtC*, which reduces the concentration of adenosine triphosphate (ATP) [15,16]. PhoP is hyperactivated by the positive feedback exerted by the MgtA and MgtC proteins when *S.* Typhimurium faces cytoplasmic Mg^2+^ starvation [17,18]. The *phoP*, *mgtB,* and *mgtC* genes are necessary for *S.* Typhimurium’s survival inside macrophages and virulence in mice [5,19,20].

Cytoplasmic Mg^2+^ starvation hinders glucose uptake by the main glucose importer PtsG of *S.* Typhimurium [2] because this stress condition dramatically suppresses CRP-cAMP amounts [2], which decreases the mRNA abundance of the CRP-cAMP-activated gene *ptsG* [21]. Replacing the wild-type *crp* gene by the constitutively active crp* allele, which specifies a cAMP-independent CRP variant due to the A144T amino acid substitution [22], increases *ptsG* mRNA abundance when *S.* Typhimurium is inside macrophages, but has little or no effect on the mRNA abundance of the glycerol kinase *glpK* gene or the gluconate transporter *gntT* gene [2] even though both *glpK* and *gntT* are also CRP-cAMP-activated genes [23,24]. By contrast, replacing the wild-type *crp* gene by a *crp* null allele encoding the inactive CRP variant with the E72A substitution hardly lowers the mRNA amounts of the three genes [2], suggesting that *S.* Typhimurium has low CRP-cAMP amounts when inside macrophages. Thus, the surprising preference of gluconate and glycerol over glucose exhibited by *S.* Typhimurium during infection [3,4] appears to result from the different sensitivities of the CRP-cAMP-activated targets responsible for the utilization of these carbon sources to a decrease in CRP-cAMP amounts.

We now report that the virulence regulatory system PhoP/PhoQ governs *S.* Typhimurium’s metabolism during cytoplasmic Mg^2+^ starvation by dictating cAMP synthesis, thereby determining the amounts of active CRP protein. We establish that the PhoP-activated *mgtA, mgtB,* and *mgtC* genes sustain a low level of cAMP production that generates enough active CRP to support diauxic growth (i.e., growth on CRP-dependent carbon sources after glucose is exhausted). By enabling utilization of CRP-dependent carbon sources, PhoP slows *S.* Typhimurium growth, advancing tolerance towards antibacterial agents [25,26].

## Results

### PhoP controls *S.* Typhimurium metabolism during cytoplasmic Mg^2+^ starvation

**Fig 1:**
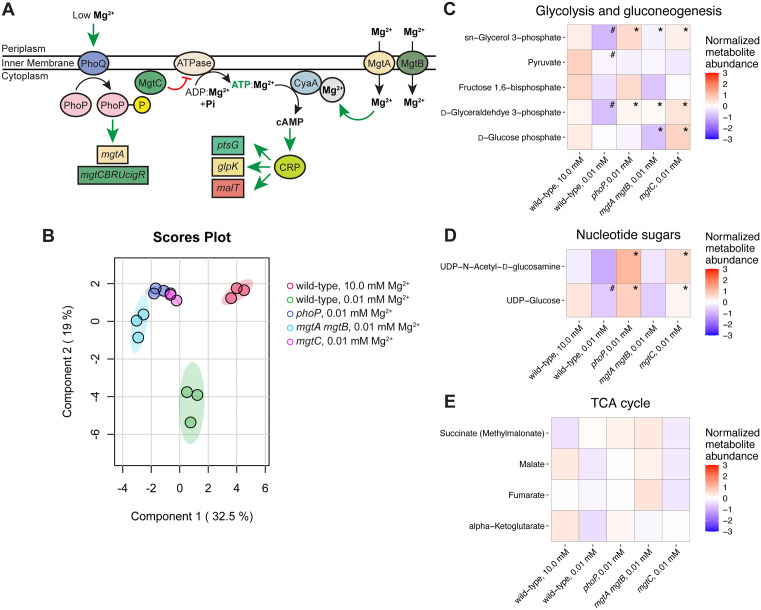
PhoP governs *S.* Typhimurium metabolism during cytoplasmic Mg^2+^ starvation. **(A)** Cartoon representation of PhoP control of CRP-cAMP. PhoP promotes transcription of the *mgtA* gene and the *mgtCBRUcigR* operon, which encode the Mg^2+^ importers MgtA and MgtB and the F_1_F_0_ ATP synthase inhibitor MgtC. Mg^2+^ is an essential cofactor of cAMP-synthesizing adenylate cyclase CyaA and ATP is CyaA’s substrate. cAMP-bound CRP directly activates transcription of the carbohydrate utilization determinants *ptsG*, *glpK*, and *malT*. Red crossbars indicate negative regulation whereas green arrows indicate positive regulation. **(B)** Dimensionality reduction by sparse partial least squares discriminant analysis (sPLS-DA) of metabolomes from isogenic wild-type (14028s), *phoP* (MS7953s), *mgtA mgtB* (EG17048), and *mgtC* (EL4) *S.* Typhimurium strains cultured in N-minimal media containing 10.0 or 0.01 mM Mg^2+^ and glycerol as carbon source. **(C–E)** Mean normalized metabolite abundance of *(C)* glycolytic, *(D)* nucleotide sugar, and *(E)* tricarboxylic acid cycle metabolites in wild-type (14028s), *phoP* (MS7953s), *mgtA mgtB* (EG17048), and *mgtC* (EL4) *S.* Typhimurium cultured in N-minimal media containing 10.0 or 0.01 mM Mg^2+^ and glycerol as carbon source. Annotations in the heatmap squares correspond to statistically significant differences in metabolite abundance between wild-type bacteria (0.01 mM vs. 10.0 mM Mg^2+^, #) or between the mutant strains and wild-type in media containing 0.01 mM Mg^2+^ (*), *p* < 0.1. *N* = 3. The data underlying this Figure can be found in S1 Data.

We hypothesized that the master regulator of virulence and Mg^2+^ homeostasis PhoP controls *S.* Typhimurium’s metabolism during cytoplasmic Mg^2+^ starvation because: first, PhoP is highly activated under this stress condition (Fig 1A) [10,17,18]. Second, the MgtA, MgtB, and MgtC proteins are anticipated to alter cAMP amounts because the cAMP-synthesizing adenylate cyclase CyaA is a Mg^2+^-dependent enzyme that uses ATP as substrate [2,27–30] and these three PhoP-activated proteins increase Mg^2+^ uptake and decrease ATP amounts (Fig 1A). And third, decreasing ATP amounts by expressing the soluble subunit of the F_1_F_0_ ATP synthase (AtpAGD) is sufficient to decrease the concentrations of both ATP and cAMP in bacteria experiencing abundant Mg^2+^, indicating that cAMP amounts are directly controlled by the availability of its immediate precursor ATP (Fig 1A) [2].

To explore the hypothesis that PhoP controls *S.* Typhimurium’s metabolism during cytoplasmic Mg^2+^ starvation, we analyzed the metabolites extracted from isogenic wild-type and *phoP* mutant *S.* Typhimurium grown for 5.0 h in N-minimal media with glycerol as carbon source and 0.01 mM Mg^2+^, a condition under which bacteria experience cytoplasmic Mg^2+^ starvation [31]. As a control, we used wild-type *S.* Typhimurium grown in media containing 10.0 mM Mg^2+^, a Mg^2+^-abundant condition [31]. To discriminate between PhoP’s effects on Mg^2+^ import versus ATP synthesis, we also included the isogenic *mgtA mgtB* double mutant and *mgtC* single mutant, respectively. (Please note that the *mgtA mgtB* double mutant has moderately higher ATP abundance than the wild-type strain during cytoplasmic Mg^2+^ starvation, but nowhere near the very high ATP abundance of the *phoP* and *mgtC* single mutants [7]).

Dimensionality reduction of metabolome composition by sparse partial least squares discriminant analysis (sPLS-DA) revealed that the metabolic alterations controlled by PhoP, MgtA, MgtB, and MgtC are similar. This is because the metabolomes of the *phoP*, *mgtA mgtB*, and *mgtC* mutants clustered together during cytoplasmic Mg^2+^ starvation (Fig 1B). Curiously, the metabolomes of the three mutants clustered more closely with the metabolome of the wild-type strain grown under Mg^2+^-abundant conditions than with the metabolome of the wild-type strain during cytoplasmic Mg^2+^ starvation (Fig 1B). In agreement with previous results [2], the metabolome of the wild-type strain grown under Mg^2+^-abundant conditions clustered independently from that corresponding to the wild-type strain during cytoplasmic Mg^2+^ starvation.

In other words, the second major component of variation (component 2, represented by the *y*-axis) distinguishes wild-type *S.* Typhimurium experiencing cytoplasmic Mg^2+^ starvation from the same strain under Mg^2+^-abundant conditions, but not from the three mutants experiencing cytoplasmic Mg^2+^ starvation (Fig 1B). Although the metabolic states of the *phoP*, *mgtA mgtB*, and *mgtC* mutants—inferred by metabolite abundance—cluster near that of wild-type *S.* Typhimurium when Mg^2+^ is plentiful (Fig 1B), the three mutants are clearly distinguished from the wild-type strain grown under Mg^2+^-abundant conditions along the first major component of variation (component 1, represented by the *x*-axis) (Fig 1B). Component 1 also explains a substantial portion of the variance between wild-type bacteria experiencing cytoplasmic Mg^2+^ starvation versus Mg^2+^-abundant conditions (Fig 1B), albeit less than the mutant strains experiencing cytoplasmic Mg^2+^ starvation (Fig 1B).

We determined that some of the metabolic changes controlled by PhoP are attributable to Mg^2+^ import by the MgtA and MgtB proteins, others to inhibition of ATP synthesis by the MgtC protein, and some to both. For example, the abundance of *sn*-glycerol 3-phosphate, a metabolite immediately downstream of glycerol in its utilization as a carbon source [32], was much lower during cytoplasmic Mg^2+^ starvation than in Mg^2+^-abundant conditions in the wild-type strain (Fig 1C), in agreement with our previous findings [2]. However, *sn*-glycerol 3-phosphate abundance was actually higher in the *phoP* and *mgtC* mutants than in the wild-type strain during cytoplasmic Mg^2+^ starvation (Fig 1C). By contrast, the *sn*-glycerol 3-phosphate abundance in the *mgtA mgtB* double mutant was similar to that of the wild-type strain (Fig 1C). These results indicate that inhibition of ATP synthesis, but not of Mg^2+^ uptake, hinders immediate glycerol utilization. (Please note that changes in the abundance of a given metabolite could reflect altered synthesis, altered consumption, or a combination of the two, which cannot be distinguished based on the available metabolomics data alone).

ATP synthesis also governs the abundance of the nucleotide sugars uridine diphosphate (UDP)-glucose and UDP-*N*-acetyl-d-glucosamine. UDP-glucose is a precursor of capsule component colanic acid and of the lipid A-modifying 4-amino-l-arabinose [33]. UDP-*N*-acetyl-d-glucosamine is a precursor of peptidoglycan and lipopolysaccharide synthesis [34,35]. The abundance of both nucleotide sugars decreased during cytoplasmic Mg^2+^ starvation in the wild-type strain but was markedly higher in the *phoP* and *mgtC* mutants (Fig 1D). By contrast, the abundance of these metabolites in the *mgtA mgtB* mutant was similar to that in wild-type bacteria under cytoplasmic Mg^2+^ starvation (Fig 1D). The same behavior was observed for *N*-acetyl-d-glucosamine phosphate, the immediate precursor of UDP-*N*-acetyl-d-glucosamine (S1A Fig).

The abundance of most detected tricarboxylic acid (TCA) cycle intermediates were moderately, albeit non-significantly, lower in the wild-type strain during cytoplasmic Mg^2+^ starvation than in Mg^2+^-abundant conditions (Fig 1E), in agreement with previous results [2]. By contrast, the *phoP* and *mgtA mgtB* mutants had higher, albeit non-significant, mean abundance of these metabolites than the wild-type strain (Fig 1E), whereas the *mgtC* mutant exhibited non-significantly higher abundance only of alpha-ketoglutarate when compared to the wild-type strain under low cytoplasmic Mg^2+^ (Fig 1E). Because carbon flux from glycerol to TCA cycle intermediates decreases during cytoplasmic Mg^2+^ starvation independently of CRP-cAMP [2], these results raise the possibility that MgtA and MgtB govern TCA cycle flux. Thus, Mg^2+^ import, but not decreased ATP synthesis, appears to govern TCA cycle metabolism during cytoplasmic Mg^2+^ starvation.

The key glycolytic intermediate d-glyceraldehyde 3-phosphate (Fig 1C) and the aromatic amino acids tryptophan and tyrosine (S1B Fig) were in lower abundance during cytoplasmic Mg^2+^ starvation than in Mg^2+^-abundant conditions in the wild-type strain, in agreement with prior results [2]. By contrast, these metabolites were in higher abundance in *phoP*, *mgtA mgtB,* and *mgtC* mutants than in the wild-type strain during cytoplasmic Mg^2+^ starvation (Figs 1C and S1B). These results indicate that PhoP controls the abundance of these metabolites both by increasing Mg^2+^ uptake and by decreasing ATP synthesis.

Together, the results in this section reveal that PhoP dictates *S.* Typhimurium metabolism under conditions experienced inside mammalian macrophages (e.g., low cytoplasmic Mg^2+^, glycerol as carbon source) by controlling Mg^2+^:ATP homeostasis through its transcriptionally activated *mgtA, mgtB,* and *mgtC* genes.

### PhoP controls the abundance of CRP-cAMP-regulated metabolites

When experiencing cytoplasmic Mg^2+^ starvation, *S.* Typhimurium reduces CRP-cAMP activity [2], which reprograms metabolism, thereby resulting in decreased abundance of key glycolytic metabolites [2]. Intriguingly, that the *phoP*, *mgtA mgtB*, and *mgtC* mutants contain higher abundance of certain glycolytic metabolites, such as *sn*-glycerol 3-phosphate and d-glyceraldehyde 3-phosphate (Fig 1C), is reminiscent of the metabolic behavior exhibited by a strain with the constitutively active crp* allele during cytoplasmic Mg^2+^ starvation [2]. These results led us to hypothesize that the altered abundance of some metabolites exhibited by wild-type *S.* Typhimurium during cytoplasmic Mg^2+^ starvation results from a PhoP-dependent decrease in CRP-cAMP amounts (Fig 1A). We tested this hypothesis by comparing metabolite abundance in wild-type *S.* Typhimurium side-by-side the crp*, phoP, mgtA mgtB, and *mgtC* mutants during cytoplasmic Mg^2+^ starvation [2,22].

**Fig 2:**
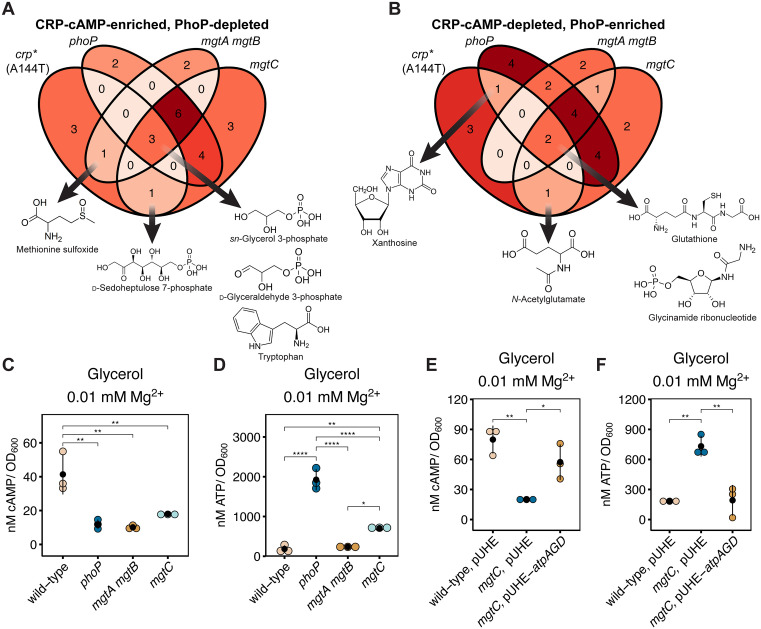
PhoP controls CRP-cAMP-regulated metabolism and cAMP synthesis during cytoplasmic Mg^2+^ starvation. **(A, B)** Venn diagram of metabolites which are *(A)* enriched by CRP-cAMP (as inferred by increased abundance in the constitutive crp* strain; metabolomics data obtained from reference [2]) and depleted by PhoP, MgtA/MgtB, or MgtC; or conversely, metabolites which are *(B)* depleted by CRP-cAMP and enriched by PhoP, MgtA/MgtB, or MgtC. **(C, D)** Intracellular cAMP *(C)* or ATP *(D)* abundance in isogenic wild-type (14028s), *phoP* (MS7953s), *mgtA mgtB* (EG17048), or *mgtC* (EL4) *S.* Typhimurium strains cultured in media containing 0.01 mM Mg^2+^ and carbon source glycerol. **(E, F)** Intracellular cAMP *(E)* or ATP *(F)* abundance in wild-type (NDP096) and isogenic *mgtC* (EL473) *S.* Typhimurium harboring the empty vector pUHE21-2::*lacI*^q^ or *mgtC* (EL473) *S.* Typhimurium harboring pUHE-*atpAGD*, expressing the soluble subunits of the F_1_F_0_ ATP synthase, AtpAGD, under the control of an Isopropyl β-d-1-thiogalactopyranoside (IPTG)-inducible promoter (MP1393). Strains were cultured in media containing 0.01 mM Mg^2+^ and glycerol as carbon source. Heterologous expression was achieved by supplementation of 1.0 mM IPTG for 2.5 h. Colored dots indicate individual replicate values, black dots indicate group mean, and error bars represent the standard deviation from the mean. *N* = 3. The data underlying this Figure can be found in S1 Data.

The abundance of multiple metabolites, including *sn*-glycerol 3-phosphate, d-glyceraldehyde 3-phosphate, tryptophan, d-sedoheptulose 7-phosphate, and methionine sulfoxide, was higher in the crp*, *phoP, mgtA mgtB,* and *mgtC* mutants than in the wild-type strain (Fig 2A). These results indicate that these metabolites are enriched by CRP-cAMP and depleted by the PhoP, MgtA, MgtB, and MgtC proteins. Moreover, some metabolites depleted in the crp* strain are also depleted in the *phoP*, *mgtA mgtB,* and *mgtC* mutants (Fig 2B); these metabolites include xanthosine, glutathione, *N*-acetylglutamate, and glycinamide ribonucleotide. That the abundance of CRP-cAMP-regulated metabolites changes concomitantly with increased PhoP activity raised the possibility of PhoP controlling *S.* Typhimurium metabolism by reducing CRP-cAMP amounts and/or by interfering with its activity. In other words, the same cytoplasmic Mg^2+^ starvation condition promoting PhoP activity simultaneously suppresses CRP activity, thereby altering an overlapping set of metabolites, strongly suggesting that *S.* Typhimurium changes metabolism under infection-relevant conditions by coordinating changes in PhoP and CRP activities.

### PhoP reduces cAMP abundance during cytoplasmic Mg^2+^ starvation

The DNA binding protein CRP requires cAMP for sequence-specific recognition of promoter elements of its regulated genes [9]. Therefore, we investigated the possibility of lower CRP activity exhibited by wild-type *S.* Typhimurium during cytoplasmic Mg^2+^ starvation than in Mg^2+^-abundant conditions stemming from *S.* Typhimurium decreasing cAMP abundance during cytoplasmic Mg^2+^ starvation [2]. PhoP could impact cAMP abundance by altering the amounts of Mg^2+^ and/or ATP because: first, PhoP governs Mg^2+^ and ATP homeostasis via the MgtA, MgtB, and MgtC proteins [7,15]. Second, CyaA is a Mg^2+^-dependent enzyme that uses ATP as substrate and requires at least 1.0 mM Mg^2+^ for full activity in vitro [30]. Third, CyaA protein amounts do not change in response to changes in cytoplasmic Mg^2+^ [2]. And fourth, ATP hydrolysis is sufficient to decrease cAMP abundance in bacteria experiencing Mg^2+^-abundant conditions [2].

We expected the *phoP* and *mgtC* mutants to have higher cAMP abundance than the wild-type strain because these mutants fail to reduce ATP amounts during cytoplasmic Mg^2+^ starvation [7,15]. By contrast, we anticipated the *mgtA mgtB* mutant to have lower cAMP abundance than the wild-type strain under low cytoplasmic Mg^2+^ because this mutant is defective in Mg^2+^ uptake [7]. Surprisingly, all three mutants had lower cAMP abundance than the wild-type strain (Fig 2C). Why, then, do the *phoP* and *mgtC* mutants have lower cAMP abundance than wild-type *S.* Typhimurium despite having higher amounts of CyaA substrate ATP (Fig 2C and 2D)?

An increase in ATP amounts can hinder rather than promote cAMP synthesis because ATP in high amounts inhibits the enzymatic activity of the purified *Escherichia coli* CyaA protein [36], and the CyaA proteins from *E. coli* and *S.* Typhimurium share 96% amino acid identity over their 848 residues. In addition, ATP could titrate essential CyaA cofactor Mg^2+^ because 85% of cellular ATP exists as Mg^2+^:ATP [37]. If the abnormally low cAMP abundance of the *mgtC* mutant is solely due to its abnormally high ATP amounts, decreasing ATP amounts in an *mgtC*-independent manner should increase cAMP abundance. As reasoned, the AtpAGD-expressing plasmid, which promotes ATP hydrolysis [2,38], decreased ATP amounts and restored cAMP abundance in the *mgtC* mutant to near wild-type levels (Fig 2E and 2F), whereas the vector control had no effect (Fig 2E and 2F). Notably, the AtpAGD-mediated restoration of cAMP abundance in the *mgtC* mutant was similar to that resulting from complementation with a wild-type copy of the *mgtC* gene (S2A and S2B Fig).

Correcting ATP abundance in the *phoP* mutant by the AtpAGD-expressing plasmid increased cAMP amounts back to the level observed in wild-type bacteria experiencing cytoplasmic Mg^2+^ starvation (S2C and S2D Fig), similar to the behavior of the *mgtC* mutant (S2A and S2B Fig). cAMP amounts were restored despite the higher ATP abundance in the *phoP* mutant compared to the *mgtC* mutant (S2B and S2D Fig). We attribute the latter behavior to possible synergy between the control of ATP abundance by the MgtA, MgtB, and MgtC proteins [7].

Together, the results in this section indicate that PhoP dictates cAMP synthesis during cytoplasmic Mg^2+^ starvation by controlling the abundance of cAMP’s precursor ATP and CyaA’s enzymatic cofactor Mg^2+^.

### PhoP governs CRP-cAMP-dependent gene transcription

CRP-cAMP controls the uptake and metabolism of various carbon sources by regulating transcription of genes encoding the corresponding transporters and metabolic enzymes [9,39]. Therefore, the increased abundance of glycolytic metabolites alongside decreased cAMP amounts exhibited by the *phoP*, *mgtA mgtB*, and *mgtC* mutants could result from PhoP altering the amounts of CRP and/or cAMP. For instance, the low CRP-cAMP amounts present in wild-type *S.* Typhimurium during cytoplasmic Mg^2+^ starvation decrease glucose uptake [2]. However, the abundance of several CRP-cAMP-controlled metabolites was higher in the *phoP*, *mgtA mgtB*, and *mgtC* mutants than in the wild-type strain (Fig 2A) even though the three mutants had lower cAMP abundance than the wild-type strain (Fig 2C). These results suggested that PhoP promotes a low level of cAMP synthesis that surpasses the threshold for CRP-cAMP-dependent catabolism of certain carbon sources, but is below the cAMP amounts required for maximal carbohydrate uptake.

Under the scenario described in the previous paragraph, increased abundance of a given metabolite would reflect decreased flux of such metabolite at a CRP-cAMP-dependent node(s) in a catabolic pathway(s). In other words, when consumption of a metabolite decreases, the abundance of that metabolite increases. For example, the GlpB and GlpD proteins, which are encoded by CRP-cAMP-activated genes, metabolize *sn*-glycerol 3-phosphate to glycerone phosphate [40–42]. Therefore, CRP-cAMP-promoted transcription of the *glpB* and *glpD* genes may generate enough GlpB and GlpD proteins for *sn*-glycerol 3-phosphate metabolism to proceed in wild-type bacteria during cytoplasmic Mg^2+^ starvation, a condition in which carbohydrate uptake is already hindered [2]. By contrast, *sn*-glycerol 3-phosphate metabolism will not proceed in the *phoP*, *mgtA mgtB*, or *mgtC* mutants because they have even lower amounts of cAMP than the wild-type strain (Fig 2C) during cytoplasmic Mg^2+^ starvation.

**Fig 3:**
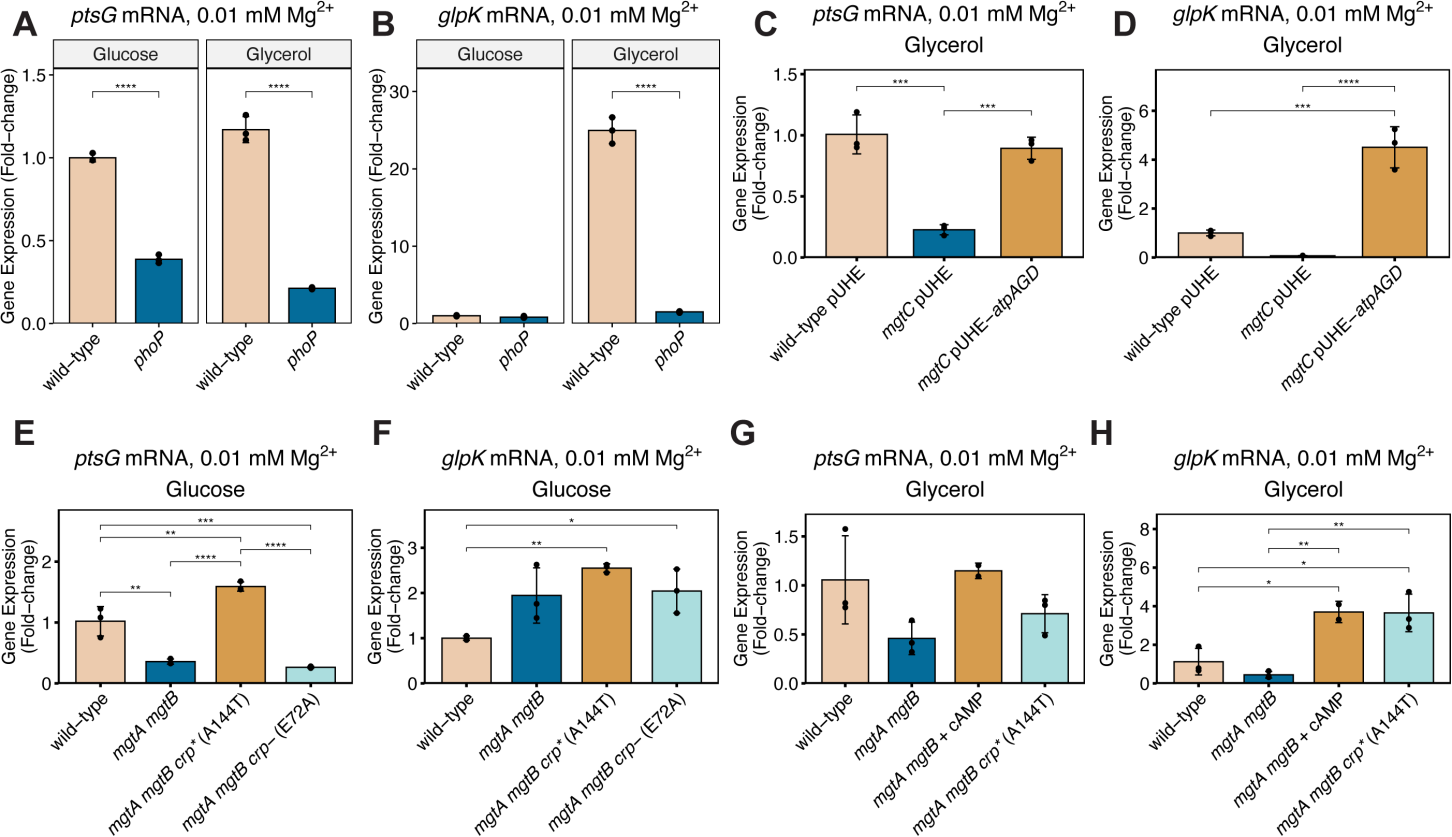
PhoP directs CRP-cAMP-dependent gene transcription during cytoplasmic Mg^2+^ starvation. **(A, B)** Relative mRNA abundance of the *ptsG (A)* and *glpK (B)* genes in isogenic wild-type (14028s) and *phoP* (MS7953s) *S.* Typhimurium strains cultured in media containing 0.01 mM Mg^2+^ and either glucose or glycerol as carbon source. Note that mRNA abundance is normalized to that in the wild-type strain under glucose-fed conditions for both the *ptsG* and *glpK* genes. **(C, D)** Relative mRNA abundance of the *ptsG (C)* and *glpK (D)* genes in isogenic wild-type (NDP096) and *mgtC* (EL473) *S.* Typhimurium strains harboring pUHE-21-2::*lacI*^q^ or *mgtC* (EL473) *S.* Typhimurium harboring pUHE-*atpAGD* (MP1393) in media containing 0.01 mM Mg^2+^ and carbon source glycerol. Heterologous expression was achieved by supplementation of 1.0 mM IPTG for 2.5 h. **(E, F)** Relative mRNA abundance of the *ptsG (E)* and *glpK (F)* genes in isogenic wild-type (14028s), *mgtA mgtB* (NDP138), mgtA mgtB crp* (A144T) (NDP143), or *mgtA mgtB crp*^*-*^ (E72A) (NDP144) *S.* Typhimurium strains cultured in media containing 0.01 mM Mg^2+^ and glucose as carbon source. **(G, H)** Relative mRNA abundance of the *ptsG (G)* and *glpK (H)* genes in isogenic wild-type (14028s), *mgtA mgtB* (NDP138), mgtA mgtB crp* (A144T) (NDP143) cultured in media containing 0.01 mM Mg^2+^ and glycerol as carbon source. In parallel, *mgtA mgtB* (NDP138) *S.* Typhimurium was cultured in the same media containing 2.5 mM exogenous cAMP. Black dots correspond to individual replicates, bars depict the group mean, and error bars represent the standard deviation from the mean. *N* = 3. The data underlying this Figure can be found in S1 Data.

To explore whether PhoP controls CRP-cAMP-dependent gene transcription during cytoplasmic Mg^2+^ starvation, we investigated the mRNA abundance of *ptsG*, the transcription of which requires CRP-cAMP in glucose-fed bacteria [21], and of *glpK*, the transcription of which is activated by CRP-cAMP in glycerol-fed bacteria [23]. First, we determined that the *phoP* mutant had lower *ptsG* mRNA amounts than the wild-type strain regardless of glucose or glycerol being the carbon source. The *phoP* mutant also exhibited decreased *glpK* mRNA amounts compared to the wild-type strain, but only when glycerol was the carbon source (Fig 3B), reflecting that glycerol is required to induce *glpK* expression by relieving repression by the glycerol-specific regulator GlpR [43]. The behavior of the *phoP* mutant was recapitulated by both the *mgtC* (Figs 3C, 3D, S3A, and S3B) and *mgtA mgtB* (Fig 3E–3H) mutants, indicating that the three PhoP-activated genes that control Mg^2+^:ATP homeostasis are necessary for full CRP-cAMP activity during cytoplasmic Mg^2+^ starvation.

Second, hydrolyzing ATP (via AtpAGD expression) rectified *ptsG* and *glpK* mRNA amounts in the *mgtC* mutant (Fig 3C and 3D), consistent with the restoration of cAMP synthesis (Fig 2E) resulting from correcting the abnormally high ATP abundance of this mutant (Fig 2F). Thus, MgtC promotes CRP-cAMP-dependent gene transcription by reducing the ATP concentration, thus promoting a low level of cAMP synthesis.

And third, if decreased cAMP synthesis is responsible for the reduced abundance of CRP-cAMP-activated mRNAs in the *phoP, mgtA mgtB,* and *mgtC* mutants, restoring CRP activity *independently* of cAMP should reverse the defect in the mutant. As reasoned, the constitutively active crp* allele corrected the mRNA amounts of the *ptsG* and *glpK* genes in the *mgtA mgtB* mutant (Fig 3E and 3F). As expected, the *crp* null allele used as negative control had no effect on the *ptsG* or *glpK* mRNA amounts of the *mgtA mgtB* mutant (Fig 3E and 3F). When grown on glucose, the crp* mgtA mgtB strain had only moderately higher *glpK* mRNA abundance than the isogenic *mgtA mgtB* strain (Fig 3F). This result likely reflects that the wild-type strain produces very low *glpK* mRNA amounts when growing on carbon source glucose (Fig 3B) and that the *mgtA mgtB* mutant and the wild-type strain produce similar *glpK* mRNA amounts (Fig 3F) (please note the different *y* axis scales in Fig 3B versus 3F).

As an independent test of our hypothesis, we supplemented the *mgtA mgtB* double mutant with cAMP, which can be taken up by bacteria and activate CRP [44]. When glycerol was the carbon source, the mRNA amounts of *ptsG* and *glpK* were higher in the *mgtA mgtB* double mutant supplemented with cAMP than in the non-supplemented strain (Fig 3G and 3H). Exogenous cAMP supplementation phenocopies the effect of introducing the crp* allele into the *mgtA mgtB* double mutant (Fig 3G and 3H) (albeit non-significantly for *ptsG*, which is *not* required for growth on glycerol, the carbon source used in these experiments).

By contrast, neither the crp* allele nor cAMP supplementation reversed the low mRNA amounts of CRP-cAMP-activated genes in the *phoP* (S3C–S3F Fig) and *mgtC* (S3G–S3J Fig) mutants. These results raise the possibility of the high ATP amounts in these mutants (Fig 2D) interfering with CRP-cAMP-dependent gene transcription (see Discussion for details). (That ATP hydrolysis corrected the *mgtC* mutant’s defects in both cAMP amounts (Fig 2E) and CRP-cAMP-dependent transcript amounts (Fig 3C and 3D) prevents the identification of the specific step at which excess ATP interferes with the activity of the CRP* protein or the utilization of exogenous cAMP. This is because correcting ATP amounts under these conditions would restore CRP-cAMP activity regardless of the introduction of the crp* allele or supplementation with cAMP.)

In sum, when wild-type *S.* Typhimurium faces cytoplasmic Mg^2+^ starvation, the regulatory protein PhoP activates the MgtA, MgtB, and MgtC proteins, which by maintaining Mg^2+^:ATP homeostasis enables a degree of CRP-cAMP-activity by promoting low-level cAMP synthesis.

### A novel faithful reporter of CRP-cAMP activity

Most investigated bacteria display an intrinsic preference for one carbon source over another if presented with two carbon sources at the same time. This preference is determined by the ease of utilization of the carbon sources, the resulting metabolic output, and the regulation of the corresponding carbon uptake and utilization systems [45–47]. In the presence of glucose and another carbon source, *S.* Typhimurium first metabolizes glucose, and after a lag phase, it metabolizes the other carbon source [48]. Known as diauxie, this growth behavior reflects the time a bacterium requires to synthesize the proteins that metabolize the non-glucose carbon source once glucose is exhausted [45]. To our knowledge, diauxie has only been examined under Mg^2+^-abundant conditions, which do not reflect the environment experienced by *S.* Typhimurium during systemic infection [5,6].

We hypothesized that low cytoplasmic Mg^2+^ and PhoP govern diauxic growth because they dictate CRP activity and also because CRP-dependent and -independent carbon sources are available in the PhoP-activating environment experienced by *S.* Typhimurium inside macrophages [3]. To explore this hypothesis, we engineered a plasmid-based reporter that faithfully captures the transient accumulation of cAMP taking place when enteric bacteria exhaust glucose and switch to a CRP-cAMP-dependent carbon source [49]. The reporter consists of the promoter of the CRP-cAMP-activated *malT* gene, which encodes the activator of the maltose utilization regulon [50], driving transcription of a promoterless *egfp* gene, which encodes the enhanced green fluorescent protein, in a medium copy number plasmid (S4A Fig). The *malT* promoter has been used to report on CRP-cAMP activity [51] because it is activated only by CRP-cAMP [52] and repressed by Mlc, the glucose phosphotransferase repressor protein [53]. The reporter plasmid—designated pCAMP (for CRP Activity from *malT* Promoter) —also harbors the promoter of the *yffH* gene, whose expression does not change across a wide array of conditions [54], driving transcription of a promoterless *tdtomato* gene, which encodes the tdTomato red fluorescent protein (see Materials and methods section for a detailed validation of the reporter).

**Fig 4:**
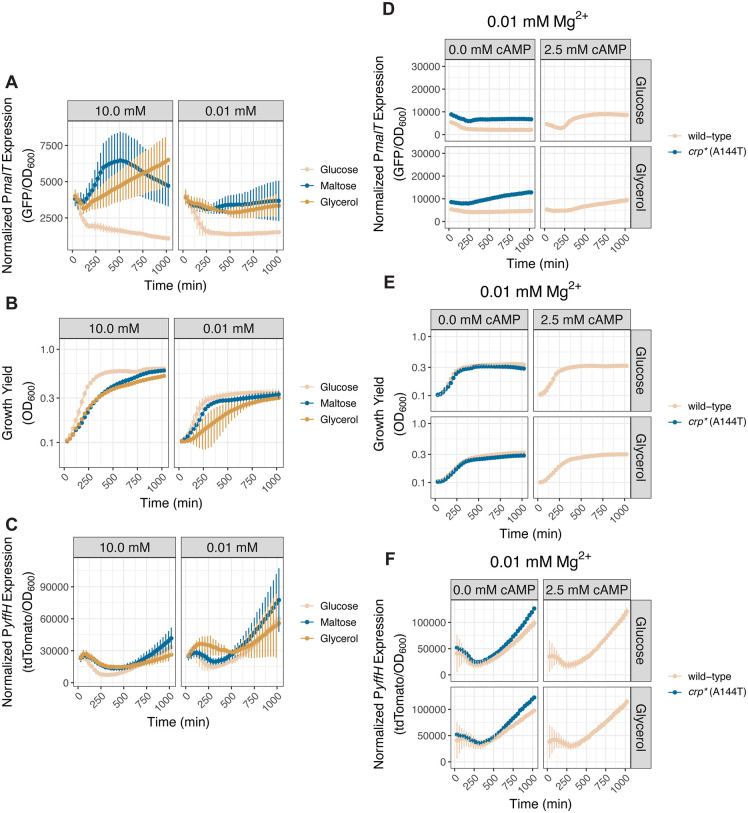
The pCAMP plasmid is a faithful reporter of CRP activity during cytoplasmic Mg^2+^ starvation. **(A–C)** *(A)* Activity of the *malT* promoter (P*malT*), *(B)* growth yield (OD_600_), and *(C)* activity of the *yffH* promoter (P*yffH*) in wild-type *S.* Typhimurium harboring the pCAMP plasmid (NDP069) cultured in media containing 10.0 or 0.01 mM Mg^2+^, glucose, maltose, or glycerol as carbon source, and casamino acids. *N* = 3. **(D–F)** *(D)* P*malT* activity, *(E)* growth yield, and *(F)* P*yffH* activity in isogenic wild-type (NDP069) and crp* (A144T) (NDP133) *S.* Typhimurium harboring the pCAMP plasmid cultured in media containing 0.01 mM Mg^2+^, glucose or glycerol as carbon source, and casamino acids. In parallel, wild-type (14028s) *S.* Typhimurium was cultured in the same media supplemented with 2.5 mM exogenous cAMP. Error bars represent the standard deviation from the mean. *N* = 2. The data underlying this Figure can be found in S1 Data.

We determined that P*malT* activity was lower during cytoplasmic Mg^2+^ starvation than under Mg^2+^-abundant conditions in wild-type *S.* Typhimurium, likely reflecting that CRP-cAMP activity is lower in the former than the latter condition [2]. The lower P*malT* activity during cytoplasmic Mg^2+^ starvation than under Mg^2+^-abundant conditions appears to be independent of carbon source because it was observed when the carbon source was glucose, glycerol, or maltose (Fig 4A).

Cytoplasmic Mg^2+^ starvation hindered bacterial growth (Fig 4B), as expected [7], and marginally increased P*yffH* activity, particularly at late timepoints when growth ceased (Fig 4B and 4C). The P*malT* activity exhibited by wild-type *S.* Typhimurium during cytoplasmic Mg^2+^ starvation is CRP-cAMP-dependent because it was absent from *crp* and *cyaA* null mutants in all tested carbon sources (S4B and S4E Fig). As expected, both *crp* and *cyaA* are required for growth on CRP-cAMP-dependent carbon sources (S4C and S4F Fig) but dispensable for P*yffH* activity (S4D and S4G Fig). (The aberrantly high P*yffH* activity of the *crp* and *cyaA* null mutants in media with casamino acids and a CRP-cAMP-dependent carbon source (S4G Fig) may result from normalization to a low OD_600_ and/or failure to dilute the tdTomato protein through cell division in non-growing bacteria.)

P*malT* activity increased during cytoplasmic Mg^2+^ starvation upon introduction of the crp* allele or cAMP supplementation (Fig 4D). However, it did not reach the levels manifested under Mg^2+^-abundant conditions (S4H Fig). These results imply that cytoplasmic Mg^2+^ regulates the amounts and/or activity of an additional factor(s) that impacts P*malT* activity (see Discussion for details). By contrast, the crp* allele or cAMP supplementation resulted in growth (Figs 4E and S4I) and P*yffH* activity (Figs 4F and S4J) similar to those of wild-type bacteria. As expected [53], inactivation of the *mlc* gene derepressed P*malT* (S4K Fig) but had no effect on growth (S4L Fig) and marginally increased P*yffH* activity (S4M Fig) during cytoplasmic Mg^2+^ starvation.

The results in this section provide independent evidence that cytoplasmic Mg^2+^ starvation decreases CRP activity. Moreover, they establish pCAMP as a faithful reporter for CRP activity under diverse growth conditions.

### Cytoplasmic Mg^2+^ controls diauxie

**Fig 5:**
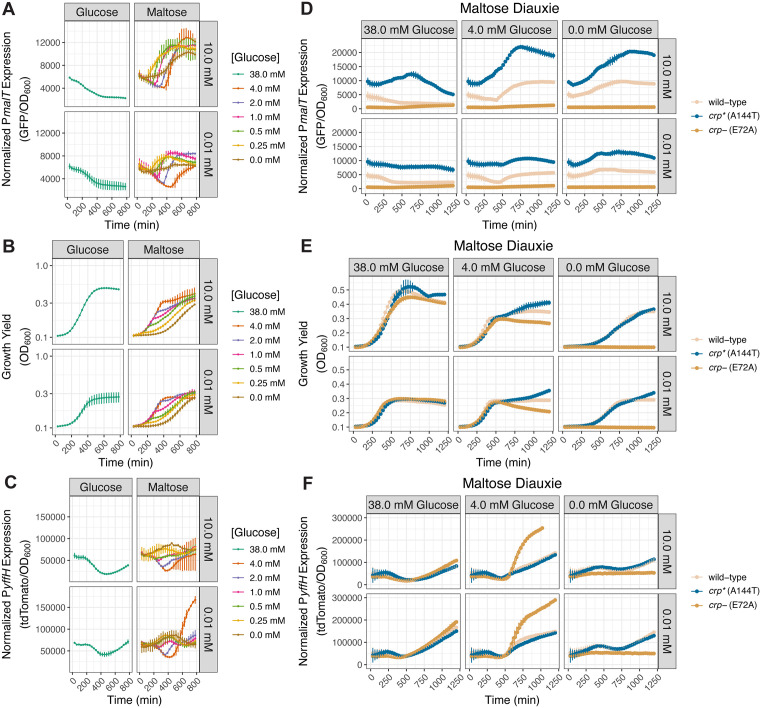
Cytoplasmic Mg^2+^ starvation governs diauxic growth. **(A–C)** *(A)* P*malT* activity, *(B)* growth yield, and *(C)* P*yffH* activity in wild-type *S.* Typhimurium harboring the pCAMP plasmid (NDP069) cultured in media containing 10.0 or 0.01 mM Mg^2+^, the indicated concentrations of glucose as primary carbon source and maltose as secondary carbon source, and lacking casamino acids. Note that the 38.0 mM glucose condition corresponds to media lacking a secondary carbon source. *N* = 2. **(D–F)** *(D)* P*malT* activity, *(E)* growth yield, and *(F)* P*yffH* activity in wild-type (NDP069), crp* (A144T) (NDP133), and *crp*^*-*^ (E72A) (NDP134) *S.* Typhimurium harboring plasmid pCAMP cultured in media containing the indicated concentrations of glucose as primary carbon source and maltose as secondary carbon source and lacking casamino acids. Error bars represent the standard deviation from the mean. *N* = 2. The data underlying this Figure can be found in S1 Data.

We sought to identify a media formulation in which *S.* Typhimurium would switch to a CRP-cAMP-dependent carbon source following glucose exhaustion while experiencing cytoplasmic Mg^2+^ starvation. Because *S.* Typhimurium experiences low cytoplasmic Mg^2+^ only after sufficiently depleting Mg^2+^ from the growth medium, at which point bacterial growth shifts from a logarithmic to linear phase [7], traditional diauxic media formulations containing only 1.0 mM glucose would result in bacteria undergoing diauxie prior to the onset of cytoplasmic Mg^2+^ starvation. Thus, we investigated P*malT* activity in media with combinations of varying glucose concentrations and a fixed (i.e., 38.0 mM) maltose concentration (Fig 5A–5C).

Wild-type *S.* Typhimurium exhibited lower P*malT* activity during the switch from glucose to maltose in media with 0.01 mM Mg^2+^ than with 10.0 mM Mg^2+^ (Fig 5A). This was true for all investigated glucose concentrations, and in agreement with the low P*malT* activity of the wild-type strain in media with 0.01 mM Mg^2+^ and a single carbon source (Fig 4A). Growth resumed poorly on maltose only when the glucose concentration was 4.0 mM and the Mg^2+^ concentration 0.01 mM (Fig 5B). That P*malT* activity was severely blunted upon switching to maltose following exhaustion of 4.0 mM glucose (Fig 5A) indicates that *S.* Typhimurium failed to synthesize enough cAMP to support growth on the CRP-cAMP-dependent maltose once it exhausted glucose.

We determined that P*malT* activity was higher and growth was better during glucose-maltose diauxie in the strain with the constitutively active crp* allele than in wild-type *S.* Typhimurium both under cytoplasmic Mg^2+^ starvation and when Mg^2+^ was abundant (Fig 5D and 5E). In agreement with these results, cAMP supplementation phenocopied the behavior of the crp* strain (S5A and S5B Fig). By contrast, there was neither P*malT* activation (Fig 5D) nor growth (Fig 5E) during glucose-maltose diauxie in the control *crp* null mutant. Also, the crp* allele (Fig 5F) and cAMP supplementation (S5C Fig) retained P*yffH* activity. Curiously, the *crp* null allele provoked aberrantly high P*yffH* activity following the shift to maltose, which we ascribe to cessation of growth of the *crp* null mutant on a CRP-dependent carbon source (Fig 5E and 5F). (See the Materials and methods section for a discussion of P*yffH* activity during diauxie (Fig 5C and 5F).)

These results establish that cytoplasmic Mg^2+^ starvation controls diauxic growth and the associated CRP-cAMP-dependent transcriptional behavior. Additionally, the results identify a media formulation in which *S.* Typhimurium undergoes diauxie while experiencing low cytoplasmic Mg^2+^.

### PhoP controls diauxie

Because PhoP governs CRP-cAMP-dependent gene transcription during cytoplasmic Mg^2+^ starvation (Fig 3), we explored the possibility of PhoP controlling the ability of wild-type *S.* Typhimurium to shift from glucose to a CRP-cAMP-dependent carbon source. Thus, we examined growth and the activities of the P*malT* and P*yffH* promoters in wild-type *S.* Typhimurium and isogenic *phoP*, *mgtA mgtB*, and *mgtC* mutants under the diauxic condition established above.

**Fig 6:**
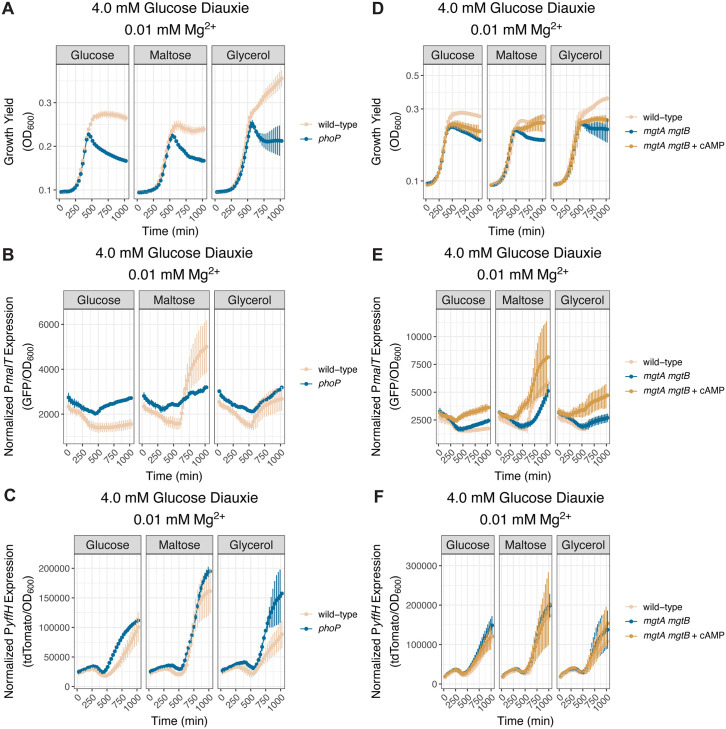
PhoP dictates diauxic growth during cytoplasmic Mg^2+^ starvation. **(A–C)** *(A)* Growth yield, *(B)* P*malT* activity, and *(C)* P*yffH* activity in isogenic wild-type (NDP069) and *phoP* (NDP070) *S.* Typhimurium strains harboring plasmid pCAMP cultured in media containing 0.01 mM Mg^2+^, 4.0 mM glucose as primary carbon source, 38.0 mM of the indicated secondary carbon source, and lacking casamino acids. *N* = 3. **(D–F)** *(D)* Growth yield, *(E)* P*malT* activity, and *(F)* P*yffH* activity in isogenic wild-type (NDP069) and *mgtA mgtB* (NDP071) *S.* Typhimurium strains harboring the pCAMP plasmid cultured in media containing 0.01 mM Mg^2+^, 4.0 mM glucose as primary carbon source, 38.0 mM of the indicated secondary carbon source, and lacking casamino acids. In parallel, *mgtA mgtB* (NDP071) *S.* Typhimurium strain was cultured in the same media supplemented with 2.5 mM exogenous cAMP. Error bars represent the standard deviation from the mean. *N* = 3. The data underlying this Figure can be found in S1 Data.

The *phoP* mutant exhibited a diminished ability to grow on maltose or glycerol following glucose exhaustion (Fig 6A), and lacked the acute P*malT* activation displayed by the wild-type strain when switching from glucose to maltose or glycerol (Fig 6B). Notably, glucose exhaustion occurred shortly after the onset of cytoplasmic Mg^2+^ starvation because the *phoP* mutant decreased growth shortly before P*malT* activation in wild-type bacteria under diauxic conditions (Fig 6A and 6B). (As we previously reported [55], the decrease in OD_600_ of the *phoP* is due to aggregation caused by cellulose accumulation to abnormally high levels.) By contrast, the *phoP* mutant had marginally higher P*yffH* activity than wild-type bacteria (Fig 6C). The *phoP* mutant displayed defective growth also in media with glucose as the sole carbon source (Fig 6A), in agreement with previous findings [56]. (We ascribe the unexpected high P*malT* and P*yffH* activity in the latter condition (Fig 6B and 6C) to normalization to the mutant’s defective growth (i.e., low OD_600_) in low cytoplasmic Mg^2+^ (Fig 6A), and also to the absence of fluorescent signal dilution through cell division [57,58] (see Materials and methods section for a detailed explanation of the normalization).)

Neither the crp* allele nor cAMP supplementation restored CRP activity to the *phoP* mutant (S3C–S3F Fig). Thus, it could not be established whether the diminished P*malT* activity manifested by the *phoP* mutant during diauxie was due to decreased CRP-cAMP amounts. Therefore, we examined the *mgtA mgtB* mutant because, like the *phoP* mutant, the *mgtA mgtB* mutant displayed diminished growth following a switch from glucose to either maltose or glycerol when compared to the wild-type strain (Fig 6D), lower P*malT* activity than the wild-type strain (Fig 6E), and unaltered P*yffH* activity (Fig 6F). Importantly, unlike the *phoP* mutant, the *mgtA mgtB* mutant does not aggregate in low cytoplasmic Mg^2+^ and responds to both the crp* allele and cAMP supplementation (Fig 3E–3H).

We determined that cAMP supplementation to the *mgtA mgtB* mutant moderately increased P*malT* activity (Fig 6E) and growth following the shift from glucose to either maltose or glycerol (Fig 6D). By contrast, cAMP supplementation to the *mgtA mgtB* mutant had no effect when glucose was the sole carbon source (Fig 6D). These results suggest that the growth defect of the *mgtA mgtB* mutant during diauxie is due, at least in part, to harboring diminished cAMP amounts, which would decrease the amounts of active CRP, thereby reducing CRP-cAMP-dependent gene transcription. Curiously, the constitutive crp* allele failed to restore growth to the *mgtA mgtB* mutant during diauxic growth (S6A Fig) even though it increased P*malT* activity (S6B Fig). As expected, the crp* allele had little effect on P*yffH* activity (S6C Fig). (The different behaviors resulting from the crp* allele and cAMP supplementation are unlikely to result from the presence of residual Mg^2+^ in the cAMP solution because cAMP supplementation actually provoked a mild growth defect during growth on glucose, whereas supplementation of additional Mg^2+^ dramatically increases growth yield (S6D Fig).) In sum, the crp* allele and cAMP supplementation can have different effects on bacterial growth despite both increasing P*malT* activity (Figs 6D and S6A; see Discussion for details).

Unlike the *phoP* and *mgtA mgtB* mutants, the *mgtC* mutant retained wild-type growth during diauxie (S6E Fig) and exhibited wild-type P*malT* (S6F Fig) and P*yffH* (S6G Fig) activities. Given that the *mgtC* mutant retains wild-type growth in media containing 0.01 mM Mg^2+^ and glucose as the sole carbon source (S6E Fig), we hypothesized that the absence of casamino acids from this medium, which slows bacterial growth [59,60], benefits the *mgtC* mutant. In agreement with this notion, the *mgtC* mutant grew poorly in 0.01 mM Mg^2+^ media containing casamino acids and glucose as carbon source (S6H Fig). Thus, conditions that slow bacterial growth alleviate the growth defects of the *mgtC* mutant.

Cumulatively, the results in this section indicate that *S.* Typhimurium’s ability to switch from glucose to a CRP-cAMP-dependent carbon source during cytoplasmic Mg^2+^ starvation requires a low level of CRP activity conferred by the PhoP-dependent transcription of the Mg^2+^ importer-encoding genes *mgtA* and *mgtB*, which promote minimal cAMP synthesis. Moreover, they establish that mutants harboring similar cAMP amounts can display different diauxic growth behaviors.

## Discussion

We have now established that the virulence regulator PhoP usurps CRP-cAMP’s reign over carbon metabolism during cytoplasmic Mg^2+^ starvation, a condition *S.* Typhimurium faces inside macrophages [5,6] that reduces CRP activity [2]. PhoP controls the amounts of active (i.e., cAMP-bound) CRP both directly, by promoting expression of the small regulatory RNA PinT that decreases CRP protein amounts [61], and indirectly, by dictating the abundance of CRP’s allosteric activator cAMP (Fig 2C and 2E). In this way, PhoP enables growth on CRP-cAMP-dependent carbon sources (Figs 4–6), albeit at a low level, causing the preferential utilization of suboptimal (i.e., non-glucose) carbon sources that results in *S.* Typhimurium’s slow growth inside macrophages [58]. Moreover, by coordinating cAMP abundance with CRP amounts, PhoP prevents the potentially detrimental effects resulting from accumulation of free (i.e., non-cAMP-bound) CRP, which can act as a nucleoid-associated protein [62].

### PhoP controls cAMP abundance by regulating cAMP synthesis

When facing cytoplasmic Mg^2+^ starvation, wild-type *S.* Typhimurium decreases, but does not eliminate, cAMP amounts, as they are higher than those of a *cyaA* mutant [2]. The cAMP produced during cytoplasmic Mg^2+^ starvation requires PhoP to promote cAMP synthesis, which PhoP accomplishes by increasing uptake of Mg^2+^ (Fig 2C), essential cofactor of the cAMP-synthesizing adenylate cyclase CyaA [30], and paradoxically, by decreasing the amounts of cAMP precursor ATP (Fig 2E). The latter favors cAMP synthesis because excess ATP both inhibits CyaA activity [36] and titrates CyaA essential cofactor Mg^2+^ [30].

In addition to PhoP’s effect on cAMP synthesis taking place under infection-relevant conditions for *S.* Typhimurium (Figs 2C and 2E), the cytoplasmic cAMP concentration could be impacted by stress and/or nutritional conditions that alter cAMP uptake, export, and/or breakdown [63,64]. For example, the cAMP increase displayed by *Mycobacterium tuberculosis* exposed to nitric oxide or acidic pH results from transcriptional repression of a gene encoding a phosphodiesterase that converts cAMP into adenosine monophosphate (AMP) by PhoP [65], a DNA-binding protein *not* homologous to the *S.* Typhimurium PhoP protein investigated in this work. Likewise, inactivation of the *fur* gene, which encodes an iron-responsive regulator [66], decreases the cytoplasmic cAMP concentration in *S.* Typhimurium grown in complex media, a decrease overcome upon mutation of the *cpdA* gene, encoding a cAMP phosphodiesterase [67].

### Disparate consequences can result from cAMP supplementation versus the constitutive crp* allele

To our knowledge, this paper and our previous study [2] are the first to report the behavior of the constitutively active, cAMP-independent crp* allele in an otherwise wild-type genetic background (i.e., previous studies examined the crp* allele exclusively in *cyaA* null genetic backgrounds [22,51,62]). We determined that the crp* allele and supplementation of exogenous cAMP can have different effects on transcriptional, metabolic, and growth behaviors when Mg^2+^ homeostasis is impaired (Figs 3E–3H and S3C–S3J). For example, both the crp* allele and exogenous cAMP restored CRP-cAMP-dependent transcription to the *mgtA mgtB* mutant (Fig 3E–3H), but not to the *phoP* or *mgtC* mutants (S3C–S3J Fig). This difference may reflect that the abnormally high ATP amounts present in *phoP* and *mgtC* mutants exacerbate cytoplasmic Mg^2+^ starvation much more than ablating Mg^2+^ uptake by the MgtA and MgtB proteins because the negatively charged ATP molecule chelates free cytoplasmic Mg^2+^ [37]. Moreover, cAMP supplementation restored growth to the *mgtA mgtB* mutant when glucose was exhausted and bacteria shifted to the CRP-cAMP-dependent carbon source (Fig 6D), but the crp* allele did not (S6A Fig). What, then, may account for these surprising behaviors?

The A144T substitution in the D-helix of the CRP* protein provokes a conformation that resembles the cAMP-bound, DNA-binding competent state of the wild-type CRP protein [22]. Therefore, the CRP* protein is locked in a conformation that may hamper control of specific genes essential for restoring growth to the *mgtA mgtB* mutant. By contrast, supplemented cAMP acts on the wild-type CRP protein to stimulate specific DNA binding by CRP-cAMP, and “excess” cAMP may be extruded and/or degraded [63,64]. In addition, cAMP catabolism to AMP by the cAMP phosphodiesterase CpdA may provide a nutritional resource to the *mgtA mgtB* mutant, whereas CRP* cannot [63].

Cytoplasmic Mg^2+^ governs the specificity of DNA-binding transcription factors, such as the eukaryotic cAMP response element binding (CREB) protein by acting as an electrostatic shield of DNA’s negatively charged phosphate backbone. This reduces transcription factor binding to non-consensus DNA without impairing CREB binding to consensus sequences [68]. We hypothesize that Mg^2+^ may similarly govern DNA binding specificity of CRP-cAMP because, in the absence of cAMP, CRP can act as a nucleoid-associated protein that governs DNA supercoiling [62]. Therefore, the exceedingly low cytoplasmic Mg^2+^ concentrations experienced by *phoP* and *mgtC* mutants (resulting from abnormally high concentrations of Mg^2+^-chelating ATP) may prevent specific CRP binding to DNA altogether, regardless of CRP being bound by cAMP. This provides a plausible explanation for the failures of the crp* allele and of excess cAMP to restore CRP-cAMP-dependent gene transcription to the *phoP* and *mgtC* mutants. Additionally, by decreasing ATP amounts, PhoP and MgtC may alter the biophysical properties of the cytoplasm, decreasing its solvent capacity because ATP solubilizes proteins in living cells [69].

### PhoP-regulated metabolic changes may increase tolerance to antibacterial agents by slowing bacterial growth

When cytoplasmic Mg^2+^ is abundant, carbon source governs metabolism, which together with amino acid availability dictates the rate of protein synthesis by governing transcription of ribosomal RNA [70,71]. However, cytoplasmic Mg^2+^ starvation supersedes these controls [2,7] (Figs 1, 4–6), slowing *S.* Typhimurium growth inside macrophages [5,6] and increasing tolerance to frontline antibiotics [25,26]. In addition to the effects on *S.* Typhimurium’s growth, cytoplasmic Mg^2+^ dictates cell envelope composition, impacting resistance to membrane-active antibacterial agents. These behaviors are coordinately controlled by PhoP, the master regulator of *S.* Typhimurium virulence and Mg^2+^:ATP homeostasis [10].

PhoP decreases the abundance of both UDP-glucose and UDP-*N*-acetyl-d-glucosamine (Fig 1D), which are metabolic precursors of several cell envelope constituents. In addition, PhoP promotes expression of some of the enzymes that use these metabolites as substrates [10]. Therefore, the cell envelope pathways favored by cytoplasmic Mg^2+^ starvation reflect both the abundance of these enzymes and their affinity for these metabolites. We propose that PhoP controls the abundance of certain metabolites, in part by consuming them in the synthesis of cell envelope constituents, some of which alter susceptibility to membrane-targeting antimicrobial agents.

Finally, the PhoP-dependent metabolic changes reported here were uncovered under infection-relevant conditions (e.g., low cytoplasmic Mg^2+^, glycerol as carbon source) that recapitulate fundamental virulence behaviors, such as hindered ATP synthesis [15], unlike in previous reports [72], as we have noted elsewhere [27]. The PhoP-dependent metabolic changes underlie slow growth and also determine antibiotic susceptibility [73,74]. Interestingly, inactivation of *cyaA* provokes heightened antibiotic tolerance in *E. coli* [75], suggesting that *S.* Typhimurium’s low CRP-cAMP amounts inside macrophages promote antibiotic tolerance. Because the PhoP protein promotes utilization of metabolically suboptimal, CRP-cAMP-dependent carbon sources during cytoplasmic Mg^2+^ starvation where glucose utilization is hindered [2], the activation of *S.* Typhimurium’s virulence program elicits physiological changes that favor slow growth, survival, and thus antibiotic tolerance in host tissues.

## Materials and methods

### Bacterial strains

All bacterial strains used in this study derive from wild-type *S.* Typhimurium strain 14028s [76]. *S.* Typhimurium was routinely cultured on lysogeny broth (LB) media unless indicated otherwise. Where appropriate, antibiotics were used at the following concentrations: 50 µg/mL ampicillin, 20 µg/mL chloramphenicol, 50 µg/mL kanamycin, and 12.5 µg/mL tetracycline. The experimental approach for evaluating bacterial behavior during cytoplasmic Mg^2+^ starvation was as follows: cultures were inoculated from a single colony grown on an appropriately selective LB agar plate into 2 mL of liquid LB broth and cultured overnight at 37 °C at 250 revolutions per minute (RPM) in a New Brunswick Scientific Innova 3100 shaking water bath. The next day, the optical density at 600 nm (OD_600_) was measured from the overnight culture, and an aliquot of cells was washed thrice in N-minimal media (5.0 mM potassium chloride, 7.5 mM ammonium sulfate, 0.5 mM potassium sulfate, 1.0 mM potassium phosphate monobasic, 50.0 mM Tris-HCl, 50.0 mM Bis-Tris, 38.0 mM glycerol, 0.1% casamino acids, pH 7.7) lacking MgCl_2_. When only one carbon source was evaluated, the wash buffer contained the relevant carbon source and casamino acids. When multiple carbon sources were compared to one another, no carbon source was added to the wash buffer. Washed cells were subcultured to an OD_600_ of 0.05 into 2.0 mL N-minimal media containing 38.0 mM of the indicated carbon source and the indicated concentration of MgCl_2_ and grown for 5.0 h at 37 °C with 250 RPM shaking in a water bath, unless stated otherwise.

For kinetic growth experiments (e.g., diauxic growth), cultures were prepared exactly as described above in N-minimal media containing the indicated concentrations of carbon sources and Mg^2+^, but lacking casamino acids where indicated. 200 µL of culture was then aliquoted in at least technical duplicate to a black glass-bottom 96-well plate and incubated in a BioTek Synergy H1 Hybrid microtiter plate reader for 24 h at 37 °C. At regular 10-min intervals, 20 s of double orbital shaking preceded fluorescence and optical density measurements. Fluorescence measurements for eGFP were acquired with 485 nm excitation wavelength, 535 nm emission wavelength, and a gain setting of 75. Fluorescence measurements for tdTomato were acquired with 550 nm excitation wavelength, 580 nm emission wavelength, and a gain setting of 134. Optical density was measured at 600 nm.

We note that growth in N-minimal media with 0.01 mM MgCl_2_ reduces the growth rate of *S.* Typhimurium after ~4.0 h of incubation [7]. For this reason, it is not possible to discriminate effects due to reduced growth rate from those associated with cytoplasmic Mg^2+^ starvation. Nevertheless, intracellular *S.* Typhimurium grow slowly due to host-mediated antibacterial mechanisms, including Mg^2+^ starvation. Slow growth in N-minimal media with 0.01 mM MgCl_2_ therefore likely recapitulates relevant behaviors of intracellular *S.* Typhimurium.

### Strain construction

Mutant *S.* Typhimurium were generated as described [77]. In brief, wild-type *S.* Typhimurium (14028s) was transformed with the temperature-sensitive pKD46 plasmid encoding the λ-red recombinase, induced with 0.2% l-arabinose for 45 min at 30 °C, and made electrocompetent by washing in sterile, ice-cold distilled water. Oligonucleotide primers were designed to amplify the chloramphenicol (Cm) or kanamycin (Km) resistance cassettes from plasmids pKD3 or pKD4, respectively, with 50 bp of sequence identity to the chromosomal site where the marker would be integrated. One microgram of PCR amplified product was electroporated into electrocompetent λ-red recombinase-expressing *S.* Typhimurium and recombinants were recovered at 37 °C in Super Optimal broth with Catabolite repression media for at least 1 h prior to being plated on appropriately selective antibiotic-containing LB agar plates and incubated overnight at 37 °C. Single colonies were screened for the appropriate insertion and loss of pKD46 by antibiotic selectivity and colony PCR with primers designed to the chromosomal site of interest. P22 lysates were prepared from positive colonies, and the mutation was transduced into a “clean” wild-type *S.* Typhimurium (14028s) background (i.e., lacking pKD46). Strains were colony purified to remove phage and screened for the absence of phage on indicator plates (8.0 g/L tryptone, 1.0 g/L yeast extract, 5.0 g/L sodium chloride, 15.0 g/L bacteriological agar, 65.0 mg/L aniline blue [Fisher Scientific A967-25], 610.0 mg/L alizarine yellow GGG LM [Sigma A6157], and 0.84% glucose).

Genomic DNA was extracted from the resultant strain to verify proper marker insertion and sequence identity by Sanger sequencing. When desired, the temperature-sensitive pCP20 plasmid harboring the yeast-derived FLP-encoding gene was transformed into the recombinant strains to excise the antibiotic resistance cassette and yield a final strain containing only the FRT scar sequence at the desired chromosomal location. Alleles from verified mutant strains were moved into different genetic backgrounds via phage P22-mediated transduction. A complete list of the strains, plasmids, and oligonucleotide primers used in this study can be found in S2, S3, and S4 Data, respectively.

### Plasmid construction

Plasmid pCAMP generated in this study (S4A Fig) was constructed using the In-Fusion homology-directed cloning kit (TakaraBio). Briefly, oligonucleotide primers were designed to linearize the pSupR plasmid backbone [54] by PCR to remove the *rdsA* promoter, generating the vector backbone. Additional oligonucleotide primers directed toward the DNA sequence corresponding to the *malT* promoter were designed with 17 bp of sequence identity to the vector backbone, generating the insert. The insert and vector were mixed at a 2:1 molar ratio (the molar ratio was increased if clones were not successfully obtained on the first pass) and added to the reaction mixture following manufacturer instructions. Positive clones were screened by colony PCR prior to plasmid purification and DNA sequencing to confirm the construct. All plasmids constructed for this study were cloned into Stellar HST08 *Escherichia coli* (TakaraBio). A complete list of plasmids and oligonucleotide primers used in this study can be found in S3 and S4 Data, respectively.

### Validation of pCAMP as a reporter of CRP-cAMP-dependent transcription

In well-aerated liquid media containing 0.01 mM Mg^2+,^ glycerol, and casamino acids, *S.* Typhimurium experiences cytoplasmic Mg^2+^ starvation after 4.0 h of growth [7]. We had to empirically determine conditions that provoked cytoplasmic Mg^2+^ starvation (as inferred by reduced CRP-cAMP activity) because a diauxic shift (defined by a lag in growth when bacteria shift from consuming one carbon source to another [1,48]) cannot be observed in the presence of casamino acids (S7A Fig), which are present in the media regularly used to examine Mg^2+^ homeostasis [78]. Note that despite the lack of a diauxic lag phase, P*malT* activity increased acutely at a time presumably corresponding to the shift from glucose to either maltose or glycerol utilization (S7B Fig), and P*yffH* activity was largely unaffected by the examined conditions (S7C Fig).

Wild-type *S.* Typhimurium harboring the reporter plasmid and cultured in Mg^2+^ abundant conditions exhibited low P*malT* transcriptional activity when grown on glucose (Fig 4A). By contrast, P*malT* activity is much higher when grown on CRP-dependent carbon sources, such as glycerol and maltose (Fig 4A). As expected, *S.* Typhimurium grew more rapidly on glucose than on maltose or glycerol (Fig 4B), and the transcriptional activity from P*yffH* remained constant across the investigated conditions (Fig 4C).

Isogenic *cyaA* and *crp* mutants failed to activate P*malT* when glucose was the carbon source in high Mg^2+^ (S4B Fig), but exhibited wild-type growth (S4C Fig), in agreement with the notion that P*malT* transcription is CRP-cAMP-dependent [52] but growth on glucose is not [1]. By contrast, the *cyaA* and *crp* mutants exhibited P*malT* activity below the level of detection (S4E Fig) and did not grow on CRP-dependent carbon source glycerol (S4F Fig) (Note that the inclusion of casamino acids in the media permitted growth following prolonged incubation (S4F Fig)). The *cyaA* and *crp* mutants retained wild-type P*yffH* transcriptional activity (S4D Fig), except in media with CRP-dependent carbon sources where no growth was observed (S4G Fig), reflecting increased accumulation of tdTomato as this protein fails to naturally dilute through cell division [57,58] and/or aberrant expression of the *yffH* promoter when growth is prohibited. cAMP supplementation restored both growth and CRP-dependent transcription to the *cyaA* mutant (S4E–S4G Fig).

The crp* allele greatly increased P*malT* transcription when Mg^2+^ was abundant, regardless of the provided carbon source (S4H Fig), but had no effect on growth (S4I Fig). Likewise, cAMP supplementation increased P*malT* activity but did not impact growth on glucose or glycerol (S4H and S4I Fig). Neither the crp* allele or cAMP supplementation had a strong effect on P*yffH* transcriptional activity under the investigated conditions (S4J Fig).

Control experiments revealed that inactivation of the *mlc* gene de-repressed P*malT* transcription when Mg^2+^ was abundant and glycerol was the carbon source (S4K Fig), but had no effect on growth (S4L Fig). By contrast, *mlc* inactivation had little effect on P*yffH* transcription (S4M Fig). Inactivation of the *mlc* gene increased P*malT* activity (S7D Fig), promoted growth on maltose (S7E Fig), but had little effect on P*yffH* activity (S7F Fig) during glucose-maltose diauxie under cytoplasmic Mg^2+^ starvation conditions, consistent with Mlc repressing P*malT* transcription and thus maltose utilization [53].

Curiously, P*yffH* activity was sensitive to the diauxic shift (Fig 5C). However, it did not resemble the sharp increase in P*malT* activity taking place during a switch to a CRP-cAMP-dependent carbon source (Fig 5A). We ascribe the unexpected P*yffH* behavior to a transient cessation of growth during the diauxic shift because P*malT* activation was still observed without a significant change in P*yffH* activity when wild-type *S.* Typhimurium was grown on a glucose-gluconate mixture (S7G–S7I Fig), in which it does not experience a diauxic shift despite gluconate utilization being CRP-cAMP-dependent [47]. Therefore, halted growth during the diauxic shift alters P*yffH* activity independently of CRP-cAMP-dependent transcription. In further support of this notion, neither the crp* allele (Fig 5F) nor cAMP supplementation (S5C Fig) changed P*yffH* activity. By contrast, P*yffH* activity was aberrantly high in a *crp*^−^ mutant, a behavior that we ascribed to its inability to grow in media with a CRP-cAMP-dependent carbon source (Fig 5E and 5F).

We utilized the *yffH* promoter as an internal normalization for the pCAMP plasmid because the *yffH* gene exhibits stable transcription across a wide variety of conditions [54]. Rather than normalize fluorescent signal from the P*malT*-driven GFP to the P*yffH*-driven tdTomato, we opted to treat these as distinct measures of activity: one which responds to the relevant experimental perturbations (e.g., CRP activity), and one that does not. This avoided the possibility of spurious results arising from changes in normalization signal, which could arise due to changes in growth or an as-yet unidentified signal that regulates *yffH* transcription.

### RNA isolation, cDNA generation, and Reverse Transcription quantitative PCR (RT-qPCR)

Approximately 0.25 OD_600_ units of cells grown in the indicated conditions were added to two volumes of RNA Protect Bacterial Reagent (QIAGEN) and incubated at RT for 10 min. Cells were collected by centrifugation and the supernatant was discarded. The cell pellets were snap frozen on dry ice and stored at −80 °C for a maximum of 2 weeks prior to RNA extraction. To extract RNA, the cell pellet was resuspended in 100 µL of Tris-EDTA (TE) buffer containing 5.0 mg/mL hen egg white lysozyme (DNA-free; Sigma Aldrich) and incubated for 10 min at RT with periodic vortexing. RNA was then isolated using the RNeasy kit (QIAGEN) following manufacturer instructions, with an on-column DNase digestion step (QIAGEN). RNA was eluted in nuclease-free water and kept at −80 °C for long-term storage.

Complementary DNA (cDNA) was generated using the SuperScript IV VILO reverse transcriptase (RT) mastermix (ThermoFisher Scientific) from 0.25-1.0 µg of total RNA (the same amount of RNA was used for all samples in each experiment) following manufacturer instructions. Prior to the RT reaction, samples were treated with ezDNase (ThermoFisher Scientific) following manufacturer instructions. cDNA was diluted 1:5 for RT-qPCR assays, and the remainder was stored at −80 °C.

RT-qPCR reactions were performed in technical triplicate using 2× Fast SYBR Green Master Mix (ThermoFisher Scientific) with 1 µL of cDNA as template on an Applied Biosystems QuantStudio6 instrument. Original primers used in this study were designed using the Integrated DNA Technologies (IDT) PrimerQuest tool and verified for >95% primer efficiency against *Salmonella* gDNA. Amplicons of interest were normalized against an amplicon targeting the *yffH* gene, the mRNA of which does not change upon environmental perturbation [54], using the ∆∆Ct method. A complete list of oligonucleotide primers used in this study can be found in S4 Data.

### Bulk metabolomics

Metabolomics sample preparation and data collection was performed by the Biological and Small Molecule Mass Spectrometry Core at the University of Tennessee Knoxville (RRID: SCR_021368). Bacteria were grown as described and collected by vacuum filtration on NucleoPore Track-Etch filters (Whatman). Frozen filtered samples prepared from 0.25–0.5 OD_600_ units of cells grown as indicated were thawed at 4 °C prior to extraction. All solvents used were LC–MS grade (Fisher Scientific). Using a previously described method [79], the extractions were performed by placing the unfolded filters cell-side down in 1.3 mL of 0.1M formic acid in 4:4:2 acetonitrile: methanol: water. The filter was allowed to extract for 20 min. The solvent was transferred to a clean microcentrifuge tube and the filter rinsed with an additional 400 µL 0.1M formic acid in 4:4:2 acetonitrile:methanol:water, and the solvents were combined. The sample suspension was then centrifuged at 17,000*g* for 5 min, and the supernatant removed. The cell pellet was re-extracted with 200 µL of the same solvent for 20 min, centrifuged for 5 min, and the supernatants combined prior to drying under a steady stream of N_2_.

The extracted and dried metabolites were resuspended in 300 µL of water prior to analysis via ultra-high performance liquid chromatography high resolution mass spectrometry. Samples were analyzed using an established untargeted metabolomics method [79] for the detection of water-soluble metabolites. Analytes were separated on a Synergi 2.6 µm Hydro-RP C18 column (100 Å, 100 mm × 2.1 mm; Phenomenex) using an UltiMate 3000 LC system (Thermo Scientific). Mobile phases consisted of 93:7 water: methanol with 15 mM acetic acid and 11 mM tributylamine as an ion pairing reagent for mobile phase A and mobile phase B was 100% methanol. The chromatographic gradient was performed as described [79]. The eluent was introduced to an Exactive Plus Orbitrap mass spectrometer (Thermo Scientific) via electrospray ionization mode in negative polarity. The mass analysis was performed in full scan mode with a scan range of 72–1,000*m*/*z* and 140,000 resolving power.

Following data collection, the Thermo.RAW files were converted to.mzML files using MSConvert from the ProteoWizard software package [80]. The.mzML files were then uploaded into an open source software package, El-MAVEN [81] (Elucidata) for peak alignment and metabolite identification and integration of peak areas. Metabolites were identified based on comparison to an in-house standard library with retention times (±2 min) and exact mass with less than 10 ppm mass error. Area under the curve (AUC) for the identified metabolites were then used for further statistical analyses. Analysis of differential metabolite abundance was performed using MetaboAnalystR [82] in RStudio, using sample normalization by sum, logarithmic data transformation, and Pareto data scaling. Statistically significant differentially abundant metabolites (*p* < 0.1) for each comparison (wild-type bacteria in 0.01 mM versus 10.0 mM Mg^2+^; *phoP*, *mgtA mgtB*, or *mgtC* mutant versus wild-type in 0.01 mM Mg^2+^; and *phoP* versus *mgtA mgtB*, *phoP* versus *mgtC*, and *mgtA mgtB* versus *mgtC* mutants in 0.01 mM Mg^2+^) are reported in S1 Data.

### ATP assay

The ATP concentration was determined using the BacTiterGlo Kit (Promega). In brief, 0.5 mL of cells grown as indicated were collected by centrifugation, washed in PBS, and resuspended in a final volume of 0.5 mL PBS, prior to heat inactivation at 70 °C for 5 min. Samples were snap-frozen on dry ice and stored at −80 °C until use. 100 µL of each sample was assayed in duplicate by combining it with 100 µL of BacTiterGlo luminescence reagent. The assay was dark-adjusted for 5 min prior to measurement of luminescence with a 1.0 s integration time in a BioTek Synergy H1 Hybrid microtiter plate reader. The ATP concentration in each sample was determined by comparison to a standard curve of ATP (Sigma Aldrich) and normalized to 1.0 OD_600_ units in 1.0 mL.

### cAMP ELISA assay

Intracellular cAMP concentration was determined using the Cyclic AMP XP Assay Kit (Cell Signaling Technologies). An OD_600_-normalized volume of culture (~0.4 OD units) grown as indicated was pelleted by centrifugation, washed in PBS to remove extracellular cAMP, and resuspended in 150 µL of lysis buffer provided with the kit. The lysis buffer was supplemented with 1 mM PMSF (Cell Signaling Technologies) prior to use. The samples were sonicated in a BioRupter Plus water bath sonicator (Diagenode) at high power with 30 s on/off cycles for 15 min. The lysate was centrifuged to remove cell debris and stored at −80 °C until use. 50 µL of lysate was assayed following the manufacturer protocol and absorbance was measured at 450 nm on a BioTek Synergy H1 Hyrbid microtiter plate reader. The intracellular cAMP concentration was determined by comparison to a standard curve of cAMP (provided with kit) and normalized to 1.0 OD_600_ units in 1.0 mL.

### Quantification and statistical analysis

All plots were generated in RStudio (R version 4.3.2, “Eye Holes”) using the packages indicated above as well as ggplot2 and derivative packages. Where applicable, black dots and error bars represent the population mean and standard deviation from the mean. Individual pairwise statistical comparisons were computed using unpaired two-tailed Tukey’s test for Honestly Significant Differences (HSD). Multiple pairwise statistical comparisons were computed by one-way ANOVA-protected unpaired two-tailed Tukey’s HSD. All tests assumed normality of the underlying data and equal variance. Unless otherwise stated, *N* = 3 for all experiments, which represents biologically and experimentally independent samples cultured, processed, and analyzed following the same procedures. Unless stated otherwise, statistical significance was defined as an adjusted *p*-value less than 0.05. * *p* < 0.05, ** *p* < 0.01, *** *p* < 0.001, **** *p* < 0.0001, ns = “not significant.

## Supporting information

S1 Data Source data file. (XLSX)

S2 Data Bacterial strains used in this study. (XLSX)

S3 Data Plasmids used in this study. (XLSX)

S4 Data Oligonucleotide primers used in this study. (XLSX)

S1 Fig PhoP governs amino sugar and amino acid metabolism during cytoplasmic Mg^2+^ starvation. **(A, B)** Mean normalized metabolite abundance of *(A)* amino sugars and derivatives and *(B)* amino acid metabolites in isogenic wild-type (14028s), *phoP* (MS7953s), *mgtA mgtB* (EG17048), and *mgtC* (EL4) *S.* Typhimurium strains cultured in N-minimal media containing 10.0 or 0.01 mM Mg^2+^ and glycerol as carbon source. *N* = 3. The data underlying this Figure can be found in S1 Data.
(TIF)

S2 Fig The PhoP-activated MgtC protein dictates cAMP synthesis during cytoplasmic Mg^2+^ starvation. **(A, B)** Determination of intracellular cAMP *(A)* or ATP *(B)* abundance in isogenic wild-type (NDP096) and *mgtC* (EL473) *S*. Typhimurium strains harboring empty vector pUHE21-2::*lacI*^q^ or *mgtC S.* Typhimurium harboring pUHE-*mgtC*, expressing the *mgtC* gene encoding an F_1_F_0_ ATP synthase inhibitor under the control of an IPTG-inducible promoter (EL474). All strains were cultured in media containing 0.01 mM Mg^2+^ and carbon source glycerol. Heterologous expression was achieved by supplementation of 0.5 mM IPTG for 2.5 h. Colored dots indicate individual replicate values, black dots indicate group mean, and error bars represent the standard deviation from the mean. *N* = 3. **(C, D)** Determination of intracellular cAMP *(C)* or ATP *(D)* abundance in isogenic wild-type (NDP096) and *phoP* (EG13135) *S.* Typhimurium strains harboring empty vector pUHE21-2::*lacI*^q^ or *phoP S.* Typhimurium harboring pUHE-*atpAGD*, expressing the soluble subunit of the F_1_F_0_ ATP synthase under the control of an IPTG-inducible promoter (NDP339). All strains were cultured in media containing 0.01 mM Mg^2+^ and carbon source glycerol. Heterologous expression was achieved by supplementation of 1.0 mM IPTG for 2.5 h. Colored dots indicate individual replicate values, black dots indicate group mean, and error bars represent the standard deviation from the mean. *N* = 3. The data underlying this Figure can be found in S1 Data.
(TIF)

S3 Fig Genetic and chemical restoration of CRP activity does not correct the transcriptional behavior of CRP-cAMP-activated genes of *phoP* and *mgtC* mutant *S.* Typhimurium strains. **(A, B)** Relative mRNA abundance of the *ptsG (A)* or *glpK (B)* genes in isogenic wild-type (14028s) or *mgtC* (EL4) *S.* Typhimurium strains cultured in media containing 0.01 mM Mg^2+^ and either glucose or glycerol as carbon source. Note that mRNA abundance is normalized to the wild-type, glucose-fed condition for both the *ptsG* and *glpK* genes. **(C, D)** Relative mRNA abundance of the *ptsG (E)* or *glpK (F)* genes in isogenic wild-type (14028s), *phoP* (MS7953s), phoP crp* (A144T) (NDP136), or *phoP crp*^*-*^ (E72A) (NDP137) *S.* Typhimurium strains cultured in media containing 0.01 mM Mg^2+^ and carbon source glucose. **(E, F)** Relative mRNA abundance of the *ptsG (E)* or *glpK (F)* genes in isogenic wild-type (14028s) or *phoP* (MS7953s) *S.* Typhimurium strains cultured in media containing 0.01 mM Mg^2+^ and glycerol as carbon source. In parallel, *phoP* (MS7953s) *S.* Typhimurium was cultured in the same media supplemented with 2.5 mM exogenous cAMP. **(G, H)** Relative mRNA abundance of the *ptsG (G)* or *glpK (H)* genes in isogenic wild-type (14028s), *mgtC* (EL4), mgtC crp* (A144T) (NDP146), or *mgtC crp-* (E72A) (NDP147) *S.* Typhimurium strains cultured in media containing 0.01 mM Mg^2+^ and carbon source glucose. **(I, J)** Relative mRNA abundance of the *ptsG (I)* or *glpK (J)* genes in wild-type (14028s) or *mgtC* (EL4) *S.* Typhimurium strains cultured in media containing 0.01 mM Mg^2+^ and glycerol as carbon source. In parallel, *mgtC* (EL4) *S.* Typhimurium was cultured in the same media supplemented with 2.5 mM exogenous cAMP. Black dots correspond to individual replicates, bars depict the group mean, and error bars represent the standard deviation from the mean. *N *= 3. The data underlying this Figure can be found in S1 Data.
(TIF)

S4 Fig The pCAMP plasmid faithfully reports on CRP activity. **(A)** Genetic map of the pCAMP plasmid (Image generated by SnapGene Viewer). **(B–G)** *(B, E)* P*malT* activity, *(C, F)* growth yield, and *(D, G)* P*yffH* activity in isogenic wild-type (NDP069), *cyaA* (NDP131), and *crp* (NDP132) *S.* Typhimurium strains harboring plasmid pCAMP cultured in media containing 10.0 or 0.01 mM Mg^2+^, *(B–D)* glucose or *(E–G)* glycerol as carbon source, and casamino acids. In parallel, *cyaA* (NDP131) *S.* Typhimurium was cultured in the same media supplemented with 2.5 mM exogenous cAMP. *N *= 2. **(H–J)** *(H)* P*malT* activity, *(I)* growth yield, and *(J)* P*yffH* activity in isogenic wild-type (NDP069) or crp* (A144T) (NDP133) *S.* Typhimurium strains harboring plasmid pCAMP cultured in media containing 10.0 mM Mg^2+^ and glucose or glycerol as carbon source. In parallel, wild-type (14028s) *S.* Typhimurium was cultured in the same media supplemented with 2.5 mM exogenous cAMP. *N *= 2. **(K–M)** *(K)* P*malT* activity, *(L)* growth yield, and *(M)* P*yffH* activity in isogenic wild-type (NDP069) or *mlc* (NDP170) *S.* Typhimurium harboring plasmid pCAMP cultured in media containing 10.0 or 0.01 mM Mg^2+^, carbon source glycerol, and lacking casamino acids. Error bars represent the standard deviation from the mean. *N *= 3. The data underlying this Figure can be found in S1 Data.
(TIF)

S5 Fig cAMP supplementation promotes CRP activity and growth during glucose-maltose diauxie. **(A–C)** *(A)* P*malT* activity, *(B)* growth yield, and *(C)* P*yffH* activity in isogenic wild-type (NDP069) and crp* (A144T) (NDP133) *S.* Typhimurium strains harboring plasmid pCAMP cultured in media containing 10.0 or 0.01 mM Mg^2+^, 4.0 mM glucose as primary carbon source, and 38.0 mM maltose as secondary carbon source, and lacking casamino acids. In parallel, wild-type (14028s) *S.* Typhimurium was cultured in the same media supplemented with 2.5 mM exogenous cAMP. Error bars represent the standard deviation from the mean. *N* = 3, except for P*yffH* activity for crp*, where *N* = 2. The data underlying this Figure can be found in S1 Data.
(TIF)

S6 Fig Mg^2+^ import, but not inhibition of the ATP synthase, governs diauxic growth. **(A–C)** *(A)* Growth yield, *(B)* P*malT* activity, and *(C)* P*yffH* activity in isogenic wild-type (NDP069) *mgtA mgtB* (NDP280), mgtA mgtB crp* (A144T) (NDP214), and *mgtA mgtB crp-* (E72A) (NDP215) *S.* Typhimurium strains harboring plasmid pCAMP cultured in media containing 10.0 or 0.01 mM Mg^2+^, 4.0 mM glucose as primary carbon source, and 38.0 mM of the indicated secondary carbon source, and lacking casamino acids. *N *= 3. **(D)** Growth yield of wild-type *S.* Typhimurium (14028s) in glucose-containing media supplemented with 0.01 or 0.05 mM Mg^2+^ in the presence or absence of 2.5 mM exogenous cAMP. *N* = 3. **(E–G)** *(E)* Growth yield, *(F)* P*malT* activity, and *(G)* P*yffH* activity in isogenic wild-type (NDP069) and *mgtC* (NDP072) *S.* Typhimurium strains harboring the pCAMP plasmid cultured in media containing 10.0 or 0.01 mM Mg^2+^, 4.0 mM glucose as primary carbon source, and 38.0 mM of the indicated secondary carbon source, and lacking casamino acids. *N *= 3. **(H)** Isogenic wild-type (NDP069) or *mgtC* (NDP072) *S.* Typhimurium strains harboring plasmid pCAMP cultured in media containing 0.01 mM Mg^2+^, carbon source glucose, and casamino acids. Error bars represent the standard deviation from the mean. *N *= 3. The data underlying this Figure can be found in S1 Data.
(TIF)

S7 Fig Validation of the pCAMP plasmid. **(A–C)** *(A)* Growth yield, *(B)* P*malT* activity, and *(C)* P*yffH* activity in wild-type *S.* Typhimurium harboring plasmid pCAMP (NDP069) cultured in media containing 10.0 or 0.01 mM Mg^2+^, 1.0 mM glucose as primary carbon source, and 38.0 mM of the indicated secondary carbon source, and casamino acids. Note the absence of a distinct diauxic shift in panel *(A)*. *N *= 2. **(D–F)** *(D)* P*malT* activity, *(E)* growth yield, and *(F)* P*yffH* activity in wild-type *S.* Typhimurium harboring plasmid pCAMP (NDP069) cultured in media containing 10.0 or 0.01 mM Mg^2+^, the indicated concentrations of glucose as primary carbon source and gluconate as secondary carbon source, and lacking casamino acids. *N *= 2. **(G–I)** *(G)* P*malT* activity, *(H)* growth yield, and *(I)* P*yffH* activity in isogenic wild-type (NDP069) and *mlc* (NDP170) *S.* Typhimurium strains harboring plasmid pCAMP cultured in media containing the indicated concentrations of glucose as primary carbon source and 38.0 mM maltose as secondary carbon source and lacking casamino acids. Error bars represent the standard deviation from the mean. *N* = 3. The data underlying this Figure can be found in S1 Data.
(TIF)

## Abbreviations

AMP: adenosine monophosphate
ATP: adenosine triphosphate
AUC: area under the curve
cAMP: 3′, 5′-cyclic adenosine monophosphate
cDNA: complementary DNA
CREB: cAMP response element binding
CRP: cAMP receptor protein
HSD: Honestly Significant Differences
LB: lysogeny broth
RPM: revolutions per minute
RT-qPCR: Reverse Transcription quantitative PCR
sPLS-DA: sparse partial least squares discriminant analysis
TCA: tricarboxylic acid
UDP: uridine diphosphate
