# Supplementary figures and images for: The master virulence regulator PhoP dictates carbon metabolism by controlling cyclic AMP synthesis in Salmonella
Source: PLoS Biol. 2025 Dec 18;23(12):e3003566. doi: 10.1371/journal.pbio.3003566 (PMC12714265; doi:10.1371/journal.pbio.3003566)

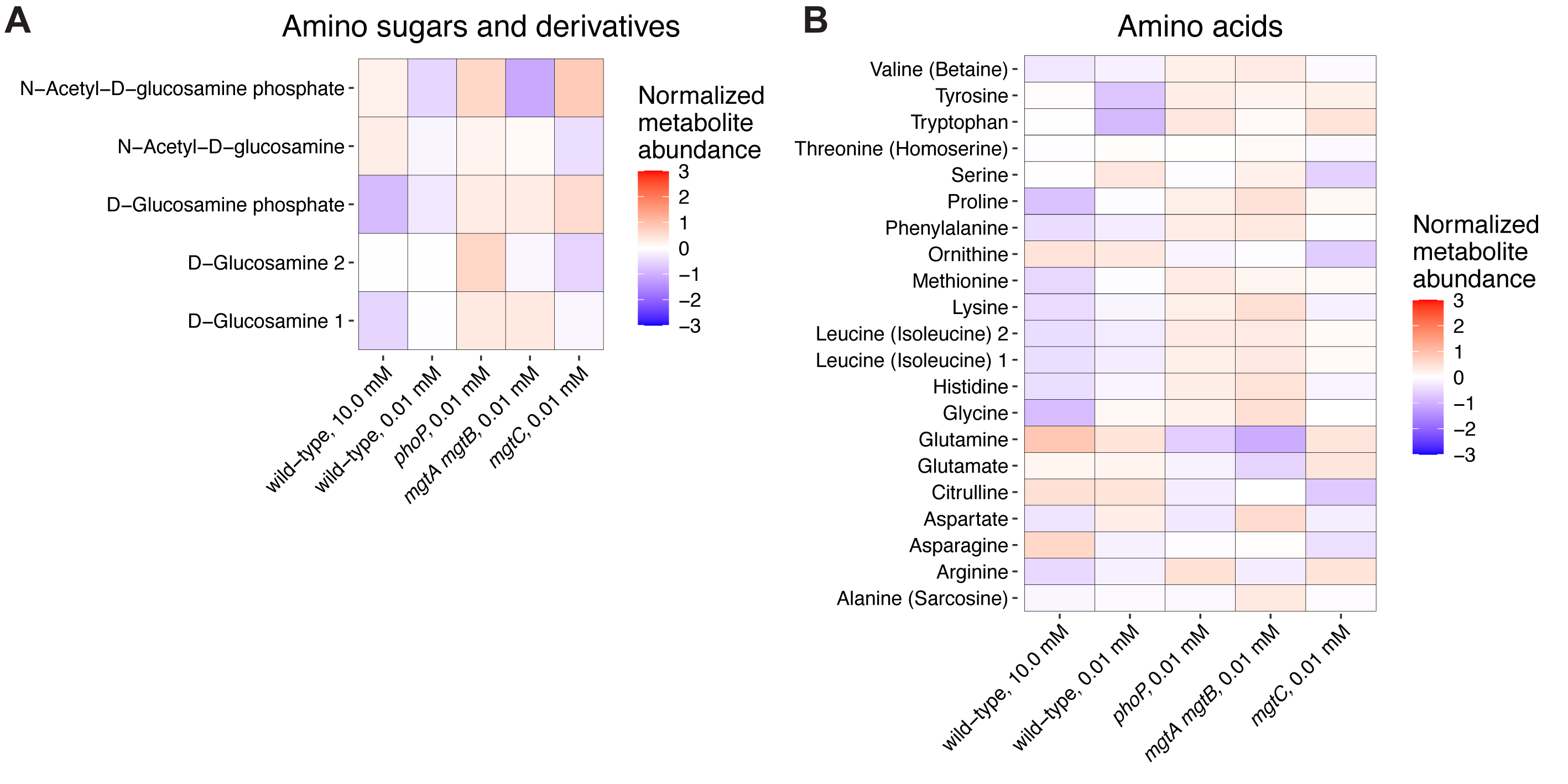

Supplement: S1 Fig — (A, B) Mean normalized metabolite abundance of (A) amino sugars and derivatives and (B) amino acid metabolites in isogenic wild-type (14028s), phoP (MS7953s), mgtA mgtB (EG17048), and mgtC (EL4) S. Typhimurium strains cultured in N-minimal media containing 10.0 or 0.01 mM Mg2+ and glycerol as carbon source. N = 3. The data underlying this Figure can be found in S1 Data. (TIF) [file pbio.3003566.s005.tif]

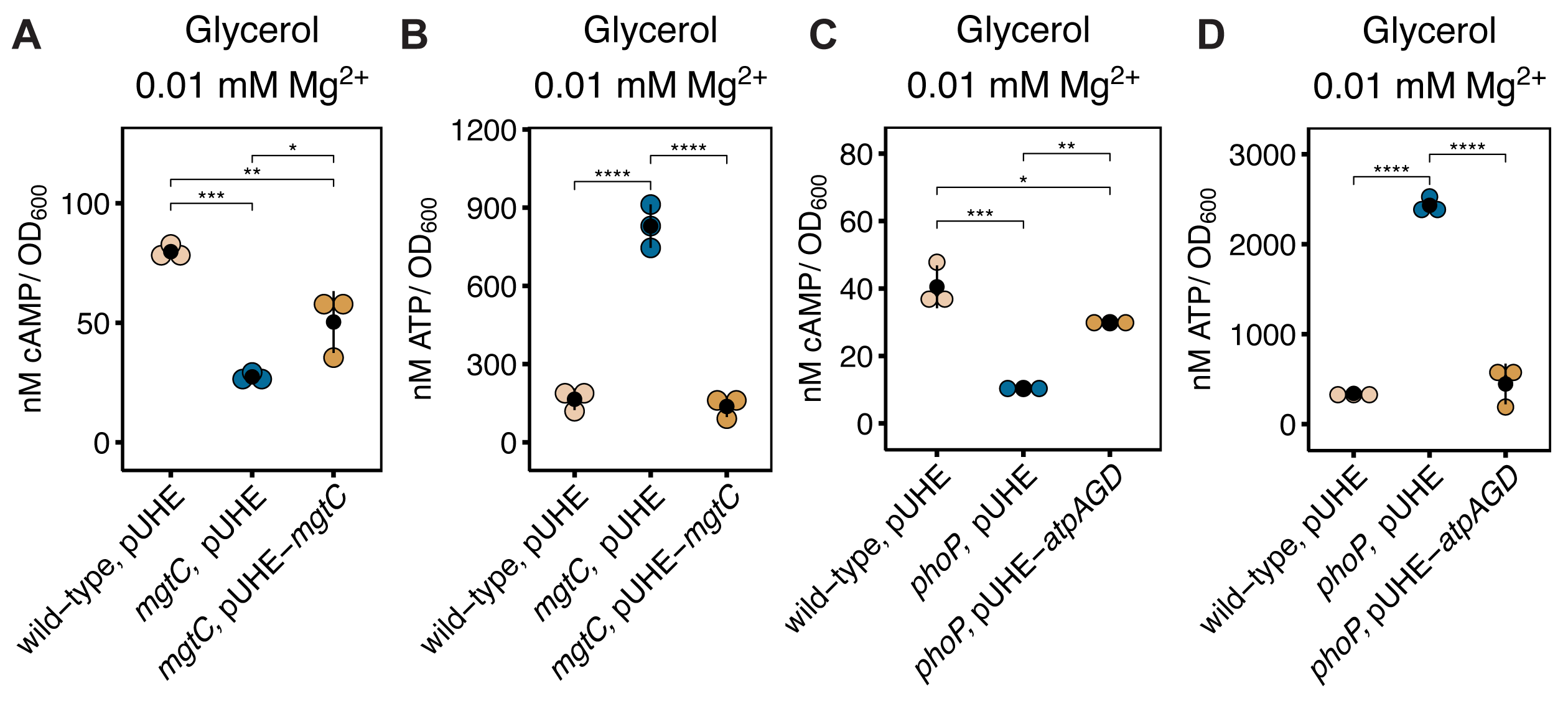

Supplement: S2 Fig — (A, B) Determination of intracellular cAMP (A) or ATP (B) abundance in isogenic wild-type (NDP096) and mgtC (EL473) S. Typhimurium strains harboring empty vector pUHE21-2::lacIq or mgtC S. Typhimurium harboring pUHE-mgtC, expressing the mgtC gene encoding an F1F0 ATP synthase inhibitor under the control of an IPTG-inducible promoter (EL474). All strains were cultured in media containing 0.01 mM Mg2+ and carbon source glycerol. Heterologous expression was achieved by supplementation of 0.5 mM IPTG for 2.5 h. Colored dots indicate individual replicate values, black dots indicate group mean, and error bars represent the standard deviation from the mean. N = 3. (C, D) Determination of intracellular cAMP (C) or ATP (D) abundance in isogenic wild-type (NDP096) and phoP (EG13135) S. Typhimurium strains harboring empty vector pUHE21-2::lacIq or phoP S. Typhimurium harboring pUHE-atpAGD, expressing the soluble subunit of the F1F0 ATP synthase under the control of an IPTG-inducible promoter (NDP339). All strains were cultured in media containing 0.01 mM Mg2+ and carbon source glycerol. Heterologous expression was achieved by supplementation of 1.0 mM IPTG for 2.5 h. Colored dots indicate individual replicate values, black dots indicate group mean, and error bars represent the standard deviation from the mean. N = 3. The data underlying this Figure can be found in S1 Data. (TIF) [file pbio.3003566.s006.tif]

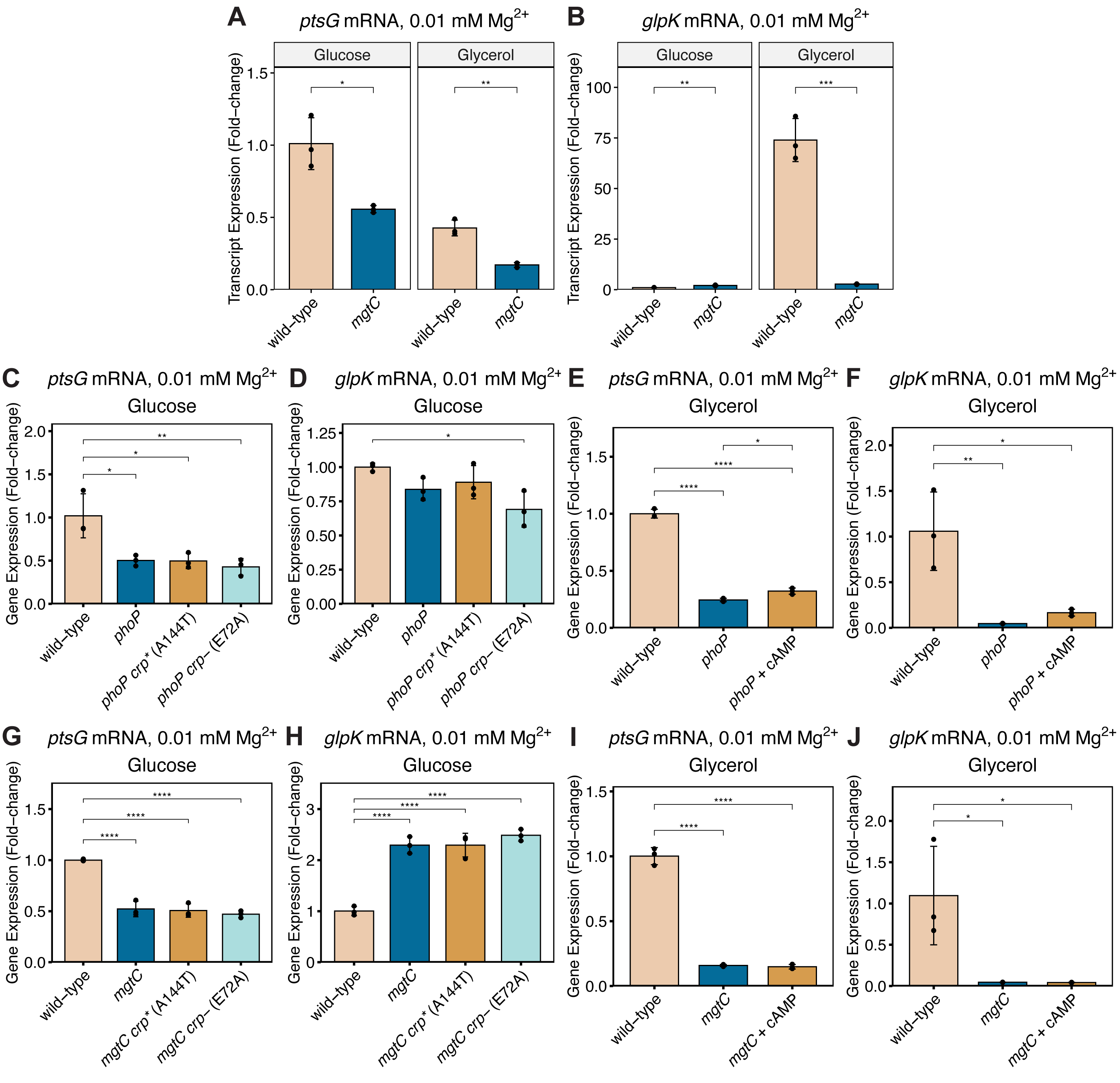

Supplement: S3 Fig — (A, B) Relative mRNA abundance of the ptsG (A) or glpK (B) genes in isogenic wild-type (14028s) or mgtC (EL4) S. Typhimurium strains cultured in media containing 0.01 mM Mg2+ and either glucose or glycerol as carbon source. Note that mRNA abundance is normalized to the wild-type, glucose-fed condition for both the ptsG and glpK genes. (C, D) Relative mRNA abundance of the ptsG (E) or glpK (F) genes in isogenic wild-type (14028s), phoP (MS7953s), phoP crp* (A144T) (NDP136), or phoP crp- (E72A) (NDP137) S. Typhimurium strains cultured in media containing 0.01 mM Mg2+ and carbon source glucose. (E, F) Relative mRNA abundance of the ptsG (E) or glpK (F) genes in isogenic wild-type (14028s) or phoP (MS7953s) S. Typhimurium strains cultured in media containing 0.01 mM Mg2+ and glycerol as carbon source. In parallel, phoP (MS7953s) S. Typhimurium was cultured in the same media supplemented with 2.5 mM exogenous cAMP. (G, H) Relative mRNA abundance of the ptsG (G) or glpK (H) genes in isogenic wild-type (14028s), mgtC (EL4), mgtC crp* (A144T) (NDP146), or mgtC crp- (E72A) (NDP147) S. Typhimurium strains cultured in media containing 0.01 mM Mg2+ and carbon source glucose. (I, J) Relative mRNA abundance of the ptsG (I) or glpK (J) genes in wild-type (14028s) or mgtC (EL4) S. Typhimurium strains cultured in media containing 0.01 mM Mg2+ and glycerol as carbon source. In parallel, mgtC (EL4) S. Typhimurium was cultured in the same media supplemented with 2.5 mM exogenous cAMP. Black dots correspond to individual replicates, bars depict the group mean, and error bars represent the standard deviation from the mean. N = 3. The data underlying this Figure can be found in S1 Data. (TIF) [file pbio.3003566.s007.tif]

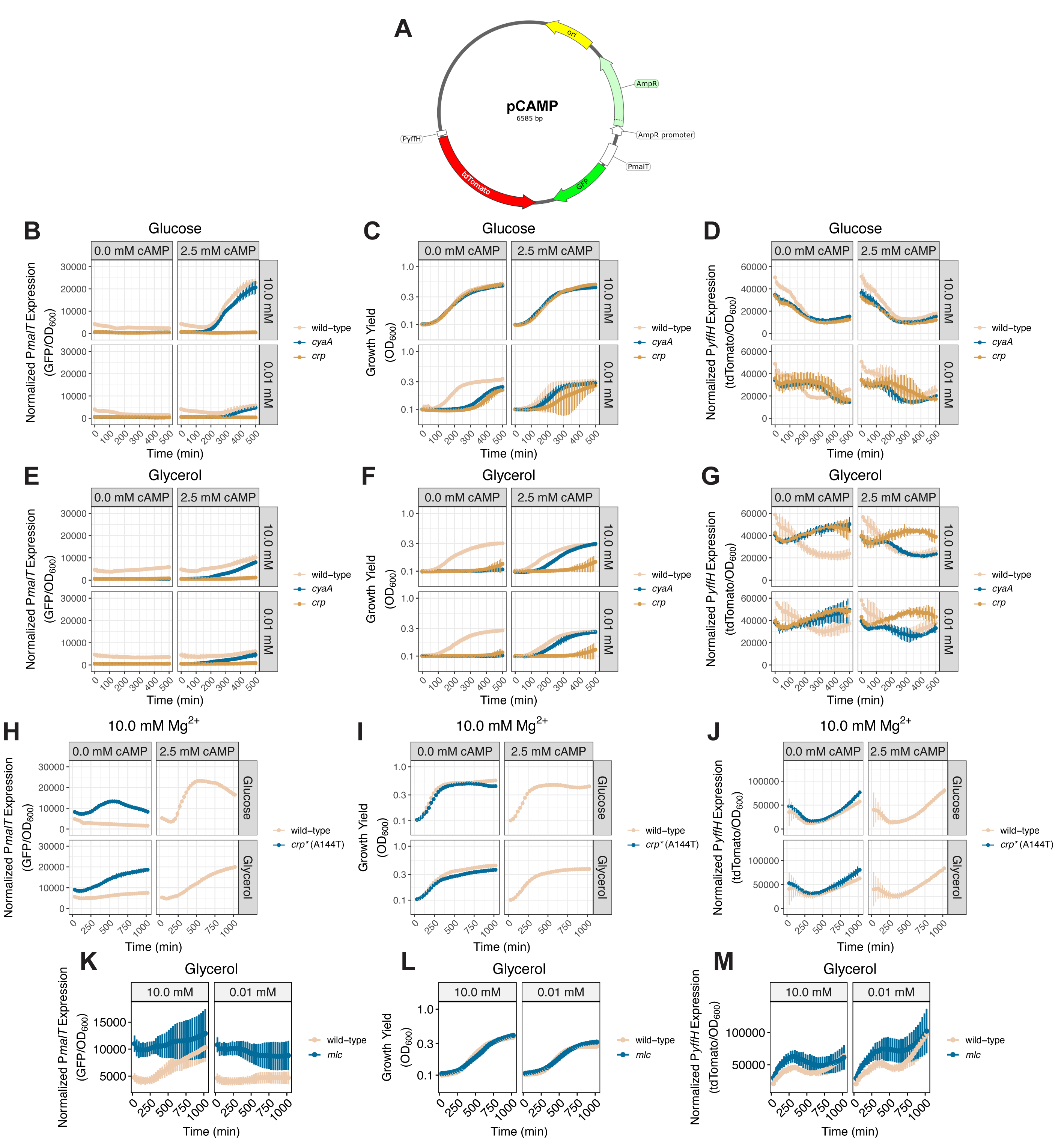

Supplement: S4 Fig — (A) Genetic map of the pCAMP plasmid (Image generated by SnapGene Viewer). (B–G) (B, E) PmalT activity, (C, F) growth yield, and (D, G) PyffH activity in isogenic wild-type (NDP069), cyaA (NDP131), and crp (NDP132) S. Typhimurium strains harboring plasmid pCAMP cultured in media containing 10.0 or 0.01 mM Mg2+, (B–D) glucose or (E–G) glycerol as carbon source, and casamino acids. In parallel, cyaA (NDP131) S. Typhimurium was cultured in the same media supplemented with 2.5 mM exogenous cAMP. N = 2. (H–J) (H) PmalT activity, (I) growth yield, and (J) PyffH activity in isogenic wild-type (NDP069) or crp* (A144T) (NDP133) S. Typhimurium strains harboring plasmid pCAMP cultured in media containing 10.0 mM Mg2+ and glucose or glycerol as carbon source. In parallel, wild-type (14028s) S. Typhimurium was cultured in the same media supplemented with 2.5 mM exogenous cAMP. N = 2. (K–M) (K) PmalT activity, (L) growth yield, and (M) PyffH activity in isogenic wild-type (NDP069) or mlc (NDP170) S. Typhimurium harboring plasmid pCAMP cultured in media containing 10.0 or 0.01 mM Mg2+, carbon source glycerol, and lacking casamino acids. Error bars represent the standard deviation from the mean. N = 3. The data underlying this Figure can be found in S1 Data. (TIF) [file pbio.3003566.s008.tif]

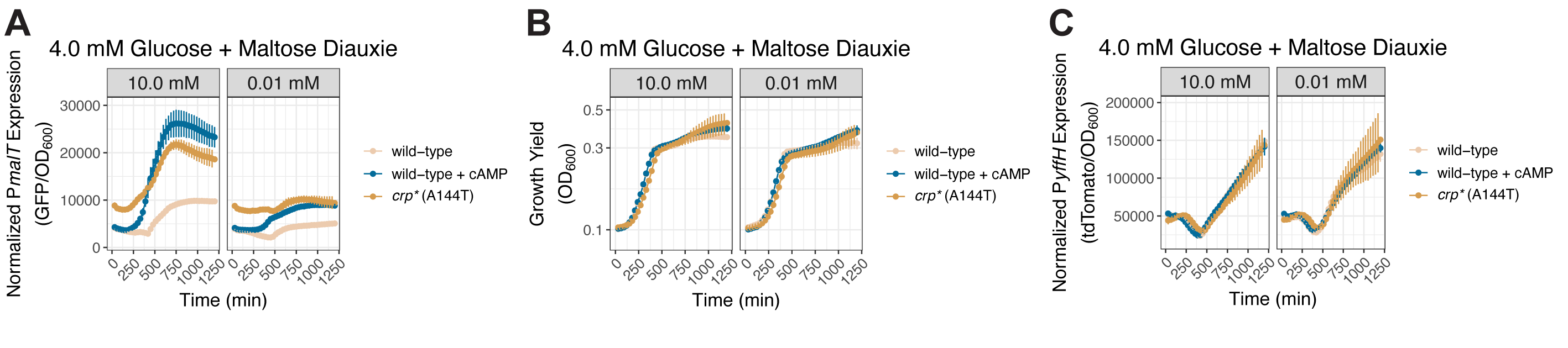

Supplement: S5 Fig — (A–C) (A) PmalT activity, (B) growth yield, and (C) PyffH activity in isogenic wild-type (NDP069) and crp* (A144T) (NDP133) S. Typhimurium strains harboring plasmid pCAMP cultured in media containing 10.0 or 0.01 mM Mg2+, 4.0 mM glucose as primary carbon source, and 38.0 mM maltose as secondary carbon source, and lacking casamino acids. In parallel, wild-type (14028s) S. Typhimurium was cultured in the same media supplemented with 2.5 mM exogenous cAMP. Error bars represent the standard deviation from the mean. N = 3, except for PyffH activity for crp*, where N = 2. The data underlying this Figure can be found in S1 Data. (TIF) [file pbio.3003566.s009.tif]

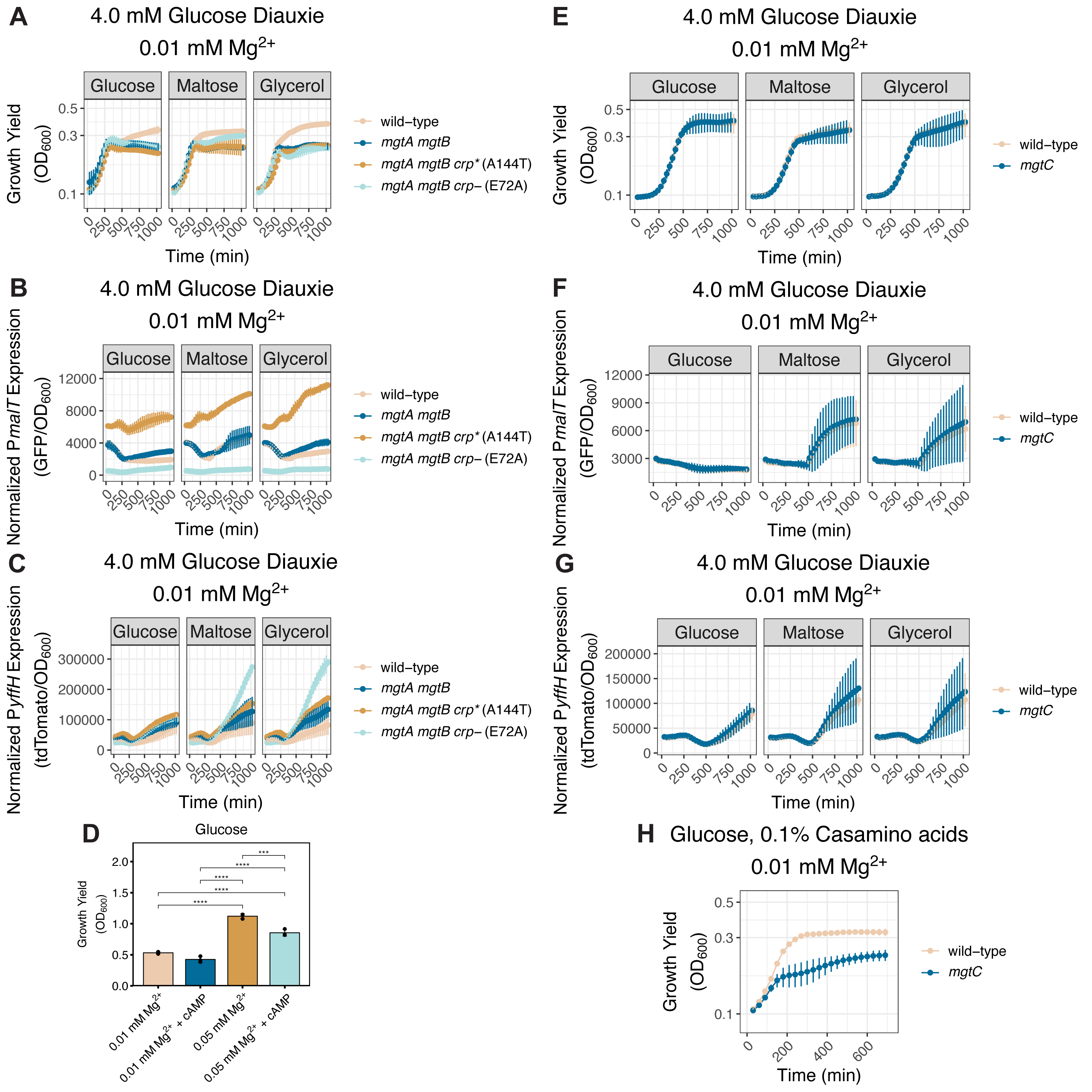

Supplement: S6 Fig — (A–C) (A) Growth yield, (B) PmalT activity, and (C) PyffH activity in isogenic wild-type (NDP069) mgtA mgtB (NDP280), mgtA mgtB crp* (A144T) (NDP214), and mgtA mgtB crp- (E72A) (NDP215) S. Typhimurium strains harboring plasmid pCAMP cultured in media containing 10.0 or 0.01 mM Mg2+, 4.0 mM glucose as primary carbon source, and 38.0 mM of the indicated secondary carbon source, and lacking casamino acids. N = 3. (D) Growth yield of wild-type S. Typhimurium (14028s) in glucose-containing media supplemented with 0.01 or 0.05 mM Mg2+ in the presence or absence of 2.5 mM exogenous cAMP. N = 3. (E–G) (E) Growth yield, (F) PmalT activity, and (G) PyffH activity in isogenic wild-type (NDP069) and mgtC (NDP072) S. Typhimurium strains harboring the pCAMP plasmid cultured in media containing 10.0 or 0.01 mM Mg2+, 4.0 mM glucose as primary carbon source, and 38.0 mM of the indicated secondary carbon source, and lacking casamino acids. N = 3. (H) Isogenic wild-type (NDP069) or mgtC (NDP072) S. Typhimurium strains harboring plasmid pCAMP cultured in media containing 0.01 mM Mg2+, carbon source glucose, and casamino acids. Error bars represent the standard deviation from the mean. N = 3. The data underlying this Figure can be found in S1 Data. (TIF) [file pbio.3003566.s010.tif]

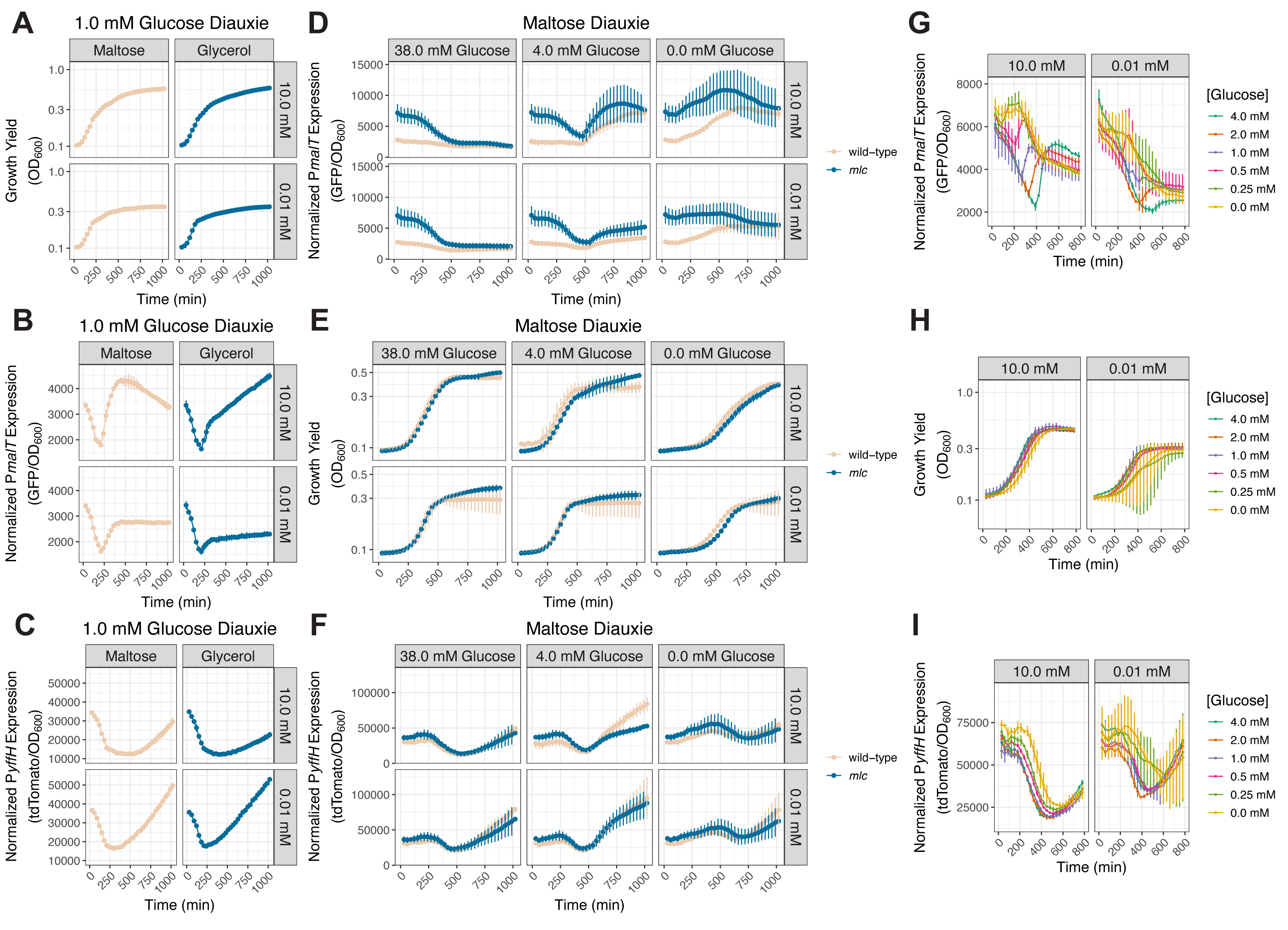

Supplement: S7 Fig — (A–C) (A) Growth yield, (B) PmalT activity, and (C) PyffH activity in wild-type S. Typhimurium harboring plasmid pCAMP (NDP069) cultured in media containing 10.0 or 0.01 mM Mg2+, 1.0 mM glucose as primary carbon source, and 38.0 mM of the indicated secondary carbon source, and casamino acids. Note the absence of a distinct diauxic shift in panel (A). N = 2. (D–F) (D) PmalT activity, (E) growth yield, and (F) PyffH activity in wild-type S. Typhimurium harboring plasmid pCAMP (NDP069) cultured in media containing 10.0 or 0.01 mM Mg2+, the indicated concentrations of glucose as primary carbon source and gluconate as secondary carbon source, and lacking casamino acids. N = 2. (G–I) (G) PmalT activity, (H) growth yield, and (I) PyffH activity in isogenic wild-type (NDP069) and mlc (NDP170) S. Typhimurium strains harboring plasmid pCAMP cultured in media containing the indicated concentrations of glucose as primary carbon source and 38.0 mM maltose as secondary carbon source and lacking casamino acids. Error bars represent the standard deviation from the mean. N = 3. The data underlying this Figure can be found in S1 Data. (TIF) [file pbio.3003566.s011.tif]
